# Sequential Ultrasound-Assisted Enzymatic Hydrolysis and Microwave Treatment of Gluten–Starch Complexes: Functionality, Volatile Profiles, In Vitro Immunoreactivity and Interactions

**DOI:** 10.3390/foods15183243

**Published:** 2026-09-14

**Authors:** Zhenlong Chen, Dong He, Xia Zhang, Lin Li, Bing Li, Xinhui Xing

**Affiliations:** 1 School of Food Science and Engineering, South China University of Technology, Guangzhou 510640, China; 2 School of Chemical Engineering and Light Industry, Guangdong University of Technology, No. 100 Waihuan Xi Road, Panyu District, Guangzhou 510006, China; 3 School of Chemical Engineering and Energy Technology, Dongguan University of Technology, College Road 1, Dongguan 523808, China; 4 Institute of Biopharmaceutical and Health Engineering, Tsinghua Shenzhen International Graduate School, Shenzhen 518055, China; 5 Key Laboratory for Industrial Biocatalysis, Ministry of Education, Institute of Biochemical Engineering, Department of Chemical Engineering, Tsinghua University, Beijing 100084, China

**Keywords:** wheat gluten, sequential processing, the Maillard reaction, gluten immunoreactivity, volatile profile, protein–starch interaction pattern

## Abstract

Sequential non-thermal and thermal processing offers a route to jointly adjust the functionality, color/volatile profile and immunoreactivity of gluten-containing systems. This study aimed to evaluate the effects of sequential ultrasound-assisted enzymatic hydrolysis, wheat/rice starch incorporation and microwave heating on soluble gluten hydrolysate–starch composites, and to probe the underlying starch–gluten interactions. Gluten hydrolysates were blended with wheat or rice starch and microwave-heated to induce the Maillard reaction. Sequential processing enriched the <1 kDa peptide fraction (2.34%→13.05–14.99%) and raised ABTS·^+^ scavenging (0.25→0.40 mM Trolox/g). After starch compounding and microwaving, the composites exhibited increased protein-normalized TEAC (0.07–0.08 to 0.10–0.13 mM per mg/mL protein) and released more ninhydrin-reactive amino groups (>55 μmol/mg protein) after in vitro digestion. All microwave composites remained pale (L* > 94), with the coupled treated rice-starch composite showing the lowest color difference (ΔE = 0.25). GC-FID/electronic-nose profiling with PCA–DFA showed that the coupled treated composites retained a native cereal-like volatile profile, marked by the green/malty aldehyde 3-methylbutanal and hexanal, with only mild roasted-type Maillard volatiles and the clearest separation for rice starch. The lowest residual R5-reactive gluten reached 8.31–9.82 ppm for the coupled treated groups. Docking provided putative models of multi-site starch binding near immunodominant gliadin sequences (e.g., QQPYP, LQPFP) via Glu/Gln–glucose hydrogen bonds and hydrophobic contacts, with more diverse binding modes for rice starch. Overall, the sequential treatment improved functional and volatile properties and lowered R5-detectable immunoreactivity for composites, offering a process reference for the development of balanced gluten-derived functional ingredients and special dietary food formulations.

## 1. Introduction

Wheat grain consists of 70–80% starch (dry weight) and 10–15% protein (dry weight) [1], of which wheat gluten accounts for about 85% [2]. It is the most widely grown cereal worldwide and a major source of energy, protein, and dietary fiber in human nutrition [3]. The properties of its two major components, gluten and starch, with multi-step food processing (i.e., mixing, baking), make wheat flour uniquely suitable for preparing a great diversity of popular baked foods [4]. Meanwhile, the Maillard reaction (MR) between the reduced sugar of starch and protein during baking contributes significantly to the desirable color and flavor of these products [5,6]. However, gluten proteins can trigger celiac disease, non-celiac wheat sensitivity, and IgE-mediated allergies such as baker’s asthma, restricting the consumption of wheat-based foods by susceptible populations [7,8]. Since native gluten is scarcely water-soluble, this limits its application in the food industry, while its aqueous hydrolysates are soluble, savory, and bioactive [9]. Therefore, recombining such water-soluble gluten fractions with starch offers a promising strategy for developing high-quality wheat-derived functional ingredients for special dietary foods that retain the nutritional and flavor-related merits of wheat-based products with reduced gluten-related risks. Moreover, wheat gluten is a globally abundant, low-cost plant protein with unique viscoelastic properties [4,10] that cannot be fully replicated by gluten-free cereals and pseudocereals [11]. Nevertheless, developing high-quality low-immunoreactive wheat protein hydrolysate-starch composite ingredients requires balancing processability, flavor, nutrition, and safety by elucidating gluten hydrolysate-starch interactions during combined processing.

Food processing can be classified as thermal processing and non-thermal processing. Thermal treatments, for example, microwave heating, offer a time- and energy-efficient route to trigger the MR [12], which under controlled conditions can concurrently enhance food sensory, nutritional, and functional attributes such as antioxidant activity [13,14]. Non-thermal technologies, including enzymatic hydrolysis (EH) for cleaving peptide bonds within linear epitopes [15], and ultrasonic treatment that remodels the conformation of allergen proteins [16], are increasingly used to mitigate the allergenicity of food matrices. However, non-thermal pretreatment alone cannot induce both heat-driven starch gelatinization and the MR, compromising texture and leaving matrices undercooked [17,18]. Thermal processing, in contrast, promotes rather than disrupts gluten covalent cross-linking and polymerization [19], thereby concealing proteolytic sites and failing to achieve sufficient hypoallergenicity.

In contrast, multi-combined processing technologies integrating thermal and non-thermal treatments have attracted increasing attention owing to their combined effects on food matrices. For example, ultrasound-assisted enzymatic hydrolysis modifies gluten structure and releases low molecular weight (Mw) peptides with enhanced antioxidant and ACE-inhibitory activities [10,20]. Incorporating exogenous proteins prior to hydrothermal treatment of starch strengthens protein–starch interactions and suppresses in vitro starch digestibility [21,22]. Enzymolysis combined with microwave heating or ultrasound can reduce the IgE immunoreactivity of food allergens [23,24]. However, each of these studies targeted only a single attribute in isolated protein, starch systems, or wheat flour (starch in wheat flour would reduce the enzyme activity [9]). Neither approach balances flavor, color, nutritional retention, and immunological safety. To date, no study has integrated ultrasound-enzymolysis pretreatment of gluten, starch incorporation, and microwave-induced MR within a single cereal-based matrix to simultaneously improve flavor quality and nutritional/functional properties and to lower the immunoreactive gluten burden. In particular, the differential effects of wheat vs. rice starch on the modified gluten system have not been systematically explored.

This study aims to sequentially integrate the combined effects of non-thermal and thermal processing to optimize the comprehensive properties of modified gluten hydrolysate-starch complexes. Specifically, wheat gluten was modified via ultrasonic pretreatment coupled with EH to avoid the interference of starch. The resulting soluble gluten hydrolysate was further blended with wheat starch (WS) or rice starch (RS) at a fixed ratio. Microwave heating was then applied to the low-moisture composite systems to induce the MR and strengthen soluble gluten hydrolysate–starch interactions. By comparing different combined processing strategies and two starch matrices, the coupled treatment was evaluated to see whether the process could simultaneously reduce gluten immunoreactivity while preserving or enhancing flavor-related quality and nutritional value. The intermolecular interactions between modified gluten hydrolysate and starch fractions were further elucidated from a multi-scale structural perspective. This work presents an innovative sequential rational design strategy (rather than a one-pot combination) for developing low-immunoreactive cereal-based functional ingredients with improved overall quality, which can serve as a supplementary component in special dietary food formulations.

## 2. Materials and Methods

### 2.1. Materials

Wheat gluten (protein content ≥ 85%, dry basis) was purchased from Heowns Biochemical Technology Co., Ltd. (Tianjin, China). WS (impurities ≤ 0.5% protein content, pH 5.0–7.0) was purchased from Shanghai Yuanye Biotechnology Co., Ltd. (Shanghai, China). RS (impurities ≤ 1% protein content, pH 5.5–7.0) was purchased from Sigma Chemical Co. (St. Louis, MO, USA). The total antioxidant capacity (T-AOC) assay kit (ABTS·^+^ method) was obtained from Beyotime Biotechnology Co., Ltd. (Shanghai, China). The amino acid (AA), ninhydrin) assay kit, Bradford protein concentration assay kit, artificial gastric juice (SGF, Chinese Pharmacopoeia (ChP)-formulated, pH 1.5, pepsin 4000 U/mL), and artificial intestinal juice (SIF, ChP-formulated phosphate buffer containing pancreatic trypsin, pH 6.8, 100 U/mL, bile-salt-free) were all obtained from Beijing Solarbio Technology Co., Ltd. (Beijing, China). Protamex compound protease (1.5 AU/g, optimal pH 5.5–7.5 at 35–60 °C) was purchased from Novozymes Inc. (Copenhagen, Denmark). The RIDASCREEN^®^ Gliadin competitive ELISA kit was purchased from R-Biopharm AG (Darmstadt, Germany), equipped with an R5 monoclonal antibody targeting immunogenic Pro/Gln-rich gluten epitopes (e.g., QQPFP, QQQFP, LQPFP and QLPFP presented in α/β-, ω- and γ-gliadins) [25]. All other reagents were of analytical grade or higher.

### 2.2. Preparation of Gluten Hydrolysates

Wheat gluten was dispersed in 200 mM phosphate-buffered saline (PBS, pH 6.0) to a final concentration of 5% (m/V) and split into three groups. (1) GO (sham-hydrolysis control): The gluten dispersion was stirred at 25 °C, 1200 rpm for 6 h, then compound protease was added at a protease-to-substrate ratio of 0.81:100 (m/m), and it was immediately inactivated at 95 °C for 10 min without effective enzymatic reaction. (2) GE (enzymatic hydrolysis): The gluten dispersion was hydrolyzed in a water bath (DF-101S, Yuhua (Gongyi), Gongyi, China) at 40 °C, 1200 rpm, pH 6.0 for 6 h with the same protease-to-substrate ratio of GO, followed by enzyme inactivation at 95 °C for 10 min. (3) GC (ultrasound-coupled enzymatic hydrolysis): The gluten dispersion (100 mL in a 250 mL beaker) was first sonicated for 30 min in a thermostatic ultrasonic bath (KQ-400GKDV, Kunshan Ultrasonic Instrument (Shumei), Kunshan, China; 40 kHz, 400 W, probe-free). Continuous stirring at 1200 rpm was applied throughout sonication using an overhead stirrer (RW 20 digital, IKA, Staufen, Germany) to improve sample homogeneity and minimize local thermal buildup, with a fixed beaker position and the sample temperature kept below 40 °C by cooling water. Then it was hydrolyzed under the same conditions as GE for 6 h, followed by enzyme inactivation [9,26]. The pH of the gluten dispersion was monitored continuously with an immersed pH probe and held near 6.0 (6.0 ± 0.1) by manual dropwise correction with dilute NaOH/HCl. After treatment, all mixtures were centrifuged (3-30KS, Sigma Laborzentrifugen, Osterode am Harz, Germany) at 9500 rpm for 20 min at 25 °C, and the supernatants were collected as soluble gluten fractions, denoted as GO, GE and GC. The soluble protein concentrations of the fractions were determined by the Bradford method and stored at 4 °C or lyophilized for subsequent analysis.

### 2.3. Gluten Hydrolysates Analysis

#### 2.3.1. Determination of Mw Distribution

Mw distribution of 1 mg/mL GO, GE, and GC in 200 mM PB (pH 6.8) was analyzed by size-exclusion high-performance liquid chromatography (SEC, Waters 600, Waters Corporation, Milford, MA, USA). The instrument was equipped with a TSK-Gel G2000SWXL or G4000SWXL column (7.8 mm i.d. × 300 mm, Tosoh Corporation, Tokyo, Japan), and detection was performed at 214 nm with a 20 μL injection. Elution was conducted at 1 mL/min using 200 mM PB (pH 6.8) as the mobile phase. Transferrin (80,000 Da), cytochrome C (12,384 Da), the synthetic peptide PVLGPVRGPH (1361 Da), glutathione (307 Da), and glutamate (147 Da) were used as standards for peak time–Mw (x-y) plotting. Each sample was fractionated into different Mw ranges (>75kDa, 75–30 kDa, 30–10 kDa, 5–10 kDa, 3–5 kDa, 1–3 kDa, and <1 kDa) based on the time ranges from the standard curve (R^2^ > 0.99). The percentage content of each Mw fragment was calculated as follows:

(1) C_i_ (%) = (A_i_/∑A_total_) × 100

where C_i_ represents the relative content of the target compound (%), A_i_ represents the integrated peak area of the target peak range, and ∑A_total_ represents the sum of all integrated peak areas of the sample. The average Mw is further determined by the following equations:

(2) M_n_ = (Σ_i_H_i_)/(Σ_i_(H_i_/M_i_))

(3) M_w_ = (Σ_i_(H_i_ × M_i_))/(Σ_i_H_i_)

where M_n_ and M_w_ are the number-average and weight-average Mw (Da), respectively; H_i_ is the detector response (integrated peak area) of the i-th elution slice, which is proportional to its mass concentration; and M_i_ is the Mw of the i-th slice, assigned from the calibration curve of standards.

#### 2.3.2. Quantitative Analysis of AAs

GO/GE/GC were pretreated via acid hydrolysis at 110 °C for 21 h. After digestion, the digestive mixture was further processed and carried out for derivatization. During derivatization, isotope-labeled internal standards were mixed in for absolute quantification. Processed samples were analyzed via an HPLC-MS/MS system (Ultimate 3000-API 3200 Q TRAP, Thermo Fisher Scientific-AB SCIEX, Marlborough, MA, USA) fitted with an MSLab HP-C18 column (150 × 4.6 mm, 5 μm). Mobile phase A was water containing 0.1% formic acid, and phase B was acetonitrile containing 0.1% formic acid. A 3 μL sample was injected and run at a 0.8 mL/min flow rate at a 50 °C column temperature. Gradient separation conditions were as follows: 2% B at 0 min, 28% B at 10 min, 100% B at 10.1 min (maintained to 16 min), and 2% B at 16.1 min (maintained to 25 min). Eluents were scanned in MRM mode via an ESI source (positive ion mode) for AA detection and quantification.

#### 2.3.3. Zeta Potential

Zeta potential of 0.1 mg/mL diluted samples in deionized water (pH 6.0, diluted from 3 mg/mL dispersed in 200 mM PBS (pH 6.0)) was determined using a Nanoparticle Zetasizer (Nano-ZS90, Malvern Instruments, Worcestershire, UK).

### 2.4. Preparation of MR Composite Products (MRCPs)

Following a preliminary single-factor screening, GO/GE/GC were thoroughly mixed with WS or RS at a 1:1 ratio. Atomized distilled water was then sprayed uniformly onto the mixed samples and mixed thoroughly to adjust the batter moisture content to ~15% (determined using a halogen moisture analyzer (HB43-S, Mettler Toledo, Zurich, Switzerland)). The wet batter was maintained at 79% relative humidity (RH, calibrated hygrometer (Testo 608-H1, Testo, Titisee-Neustadt, Germany)). The moist blended composites were preheated in a calibrated multi-mode microwave reactor (MARS 6, CEM, Matthews, NC, USA) at 600 W for 5 min. After preheating, they were further heated by microwave for 5 min at 30 s intervals for 3 repeated runs in total to produce MRCPs (denoted as HGOWS, HGEWS, HGCWS, HGORS, HGERS, HGCRS). A rotating sample turntable (5 rpm) was used to ensure uniform microwave exposure across all samples. For each treatment, 2.0 g of sample was evenly spread to an identical thickness. Three independent replicates were randomly placed in the cavity, and endpoint surface temperature was monitored to ensure deviations < ±2 °C. Microwave-prepared MRCPs or their water-soluble extracts (obtained with deionized water) were lyophilized for subsequent analyses.

### 2.5. Browning Degree and Antioxidant Ability of Scavenging ABTS·^+^

MR intermediate products (MRIPs) and MR end products (MREPs) reflect the different stages’ extent of the MR. The protein/hydrolysate–starch mixtures were dissolved in 10 mM PBS (pH 7.0) to a concentration of 3 mg/mL and then diluted 10 times. Absorbance was measured at 294 nm (for MRIPs) and 420 nm (for MREPs). ABTS·^+^ scavenging activity was determined for all samples (GO, GE, GC, HGOWS, HGEWS, HGCWS, HGORS, HGERS, HGCRS) following the T-AOC Assay Kit instructions using a microplate reader (Infinite 200 PRO, Tecan, Männedorf, Switzerland). The prepared ABTS·^+^ working solution was diluted 49-fold to an absorbance of 0.7 ± 0.05 at 734 nm and allowed to stand for 12–16 h. Samples were dissolved in deionized water to 5 mg/mL. A 10 μL aliquot of each sample solution was mixed with 200 μL of the ABTS·^+^ working solution, and absorbance was measured at 734 nm. Scavenging activity was calculated using a standard curve generated with Trolox at concentrations of 0.15, 0.3, 0.6, 0.9, 1.2, and 1.5 mM. TEAC was calculated as follows:

(4) TEAC (mM TE/(mg/mL protein)) = [(∆A_sample_ − b)/a × DF]/C_prot_

where ∆A_sample_ represents the blank-corrected absorbance of samples, and a and b are the slope and intercept of the Trolox calibration curve, respectively. DF is the sample dilution factor, and C_prot_ indicates the protein concentration of samples (mg/mL).

### 2.6. In Vitro Sequential Digestion

In vitro digestion used a static two-step (gastric-intestinal) model aligned with the INFOGEST consensus [27,28], with minor endpoint-specific modifications and ChP-formulated simulated fluids. Briefly, 30 mg of each original composite or MRCP was dispersed in 15 mL SGF (pH 1.5, pepsin 4000 U/mL) and incubated at 37 °C with shaking at 150 rpm for 2 h. A 1 mL aliquot was taken at the end of the gastric phase. Then 15 mL SIF (pancreatic trypsin, pH 6.8, 100 U/mL) was added, the pH was adjusted to 6.8, and intestinal digestion proceeded for another 4 h (37 °C, 150 rpm), with a final aliquot at 6 h total. The 4 h intestinal phase was chosen to allow cleavage of the digestion-resistant, Pro/Gln-rich gluten peptides [29]. Enzymes were inactivated at 80 °C for 20 min, and digesta were centrifuged (12,000 rpm, 5 min). Ninhydrin-reactive free α-amino groups in the supernatant were determined with the amino acid microplate kit. Centrifuged samples were mixed as per kit instructions and incubated in a boiling water bath for 15 min. Solutions were inverted repeatedly after cooling. After centrifugation (8000 rpm, 5 min), absorbance of the blue-purple compound formed in the collected supernatant was measured at 570 nm using a microplate reader (Infinite 200 PRO, Tecan, Switzerland). All measurements were completed within 30 min of color development. The detection mechanism and calculation (5) were as follows:

$$\mathrm{AAs}+\mathrm{Ninhydrin}\overset{H^{+}}{\to }\mathrm{Ruhemann}’s\;\mathrm{Purple},\;\lambda =570\;\mathrm{nm}$$

(5) C_AA_ = (10 × (A_sam_ − A_blk_))/((A_std_ − A_blk_) × C_prot_)

where C_std_ represents the standard AA concentration (10 μmol/mL); AA content (μmol/mg protein) is the digestion-released AAs measured from the supernatant; V_std_ and V_sam_ are the volumes of standard solution and volume of sample supernatant added to the reaction system, which equal 0.05 mL; C_prot_ represents supernatant protein concentration (mg/mL); and A_sam_, A_std_, and A_blk_ denote the absorbance of the sample, standard, and blank tubes, respectively. The constant value 10 is derived from C_std_ × V_std_.

### 2.7. Determination of Color Parameters

MRCPs were dissolved in distilled water to a concentration of 5 mg/mL and accurately pipetted into colorimetric cuvettes to ensure a consistent height. Before color photography, the electronic eye instrument (Digieye, VeriVide, Enderby, UK) was calibrated with white tiles and swatches. A fixed diameter was set to measure color parameters, including lightness (L: 0 = black, 100 = white), red–green value (a: +a = red, −a = green), yellow–blue value (b: +b = yellow, −b = blue), whiteness (W*), chroma (C*), and color difference (∆E*). W*, C*, and ∆E* were calculated as follows [30]:

(6) W* = 100 − [(100 − L)^2^ + a^2^ + b^2^]^1/2^

(7) C* = (a^2^ + b^2^)^1/2^

(8) ∆E* = [(L − L_0_)^2^ + (a − a_0_)^2^ + (b − b_0_)^2^]^1/2^

where L_0_, a_0_, and b_0_ represent the color parameters of control samples (without microwave heating) at t = 0.

### 2.8. Ultra-Fast Gas Chromatography (GC-FID)-Electronic Nose Determination

An ultra-fast GC electronic nose system (Heracles II, Alpha MOS., Toulouse, France) was used to detect and analyze the fingerprint profiles of flavor volatile components and differences among various composites. The system was equipped with an autosampler (Odor Scanner HS 100, Alpha M.O.S., Toulouse, France) with two parallel metal columns (non-polar MXT-5-FID1 column (10 m × 180 μm × 0.4 μm) and medium polarity MXT-1701-FID2 column (10 m × 180 μm × 0.4 μm)), a cooled Tenax trap, and two ultra-sensitive FID detectors. A 1 g aliquot of the 5 mg/mL MRCP solution was weighed into a crimp-top GC vial for detection. The vials were shaken at 50 °C for 30 min at 500 r/min to generate headspace sample gas. A volume of 3000 μL was injected at a speed of 125 μL/s and a duration of 29 s with a 200 °C inlet temperature. The initial temperature and desorption temperature of the trap were 50 °C and 240 °C, with a 34 s trapping duration, while the FID temperature was 260 °C. Compounds with a library relevance score above 30 were tentatively identified by dual-column retention-index (RI) matching against the built-in AroChemBase library (Alpha M.O.S., Toulouse, France). Odor descriptors (Supplementary Table S1) were assigned from the sensory attributes reported in AroChemBase and cross-checked against Fenaroli’s Handbook of Flavor Ingredients [31] and the Good Scents Company database (https://www.thegoodscentscompany.com/); their occurrence in cereal-baked and other thermally processed foods was interpreted following previous reports [32,33]. Three independent acquisitions were performed per treatment (*n* = 3). The peak-area/relative-content fingerprints were auto-scaled and analyzed in the AlphaSoft V12.4 workstation (built-in AroChemBase). PCA, which requires no prior labels, was used for unsupervised visualization of intrinsic clustering, whereas DFA, a supervised method that maximizes the between-/within-group variance ratio, was used to test treatment separation within each starch matrix [34,35] and was checked internally by leave-one-out cross-validation (each acquisition was held out in turn, the model refitted, and the held-out sample reassigned). DFA served as an exploratory within-dataset discrimination rather than a classifier for unknown samples.

### 2.9. Determination of Gluten Content in Composites (ELISA Assay)

MRCPs (1 g) were added to 10 mL of 60% ethanol, vortexed for ~10 min, and centrifuged at 10,000 rpm for 10 min. Supernatants were diluted 50-fold with dilution buffer. A 50 μL aliquot of diluted supernatant was dispensed into separate wells of a 96-well plate, mixed with 50 μL antibody–enzyme conjugates (stock diluted 1:11), and incubated for 30 min. The plate was inverted and blotted dry on filter paper. Each well was then washed ≥ 2 times with 250 μL of washing diluent (1:10 dilution of stock solution), with blotting dry on filter paper after each wash. A 100 μL aliquot of substrate/chromogen was added to each well and incubated for 10 min in the dark. The reaction was terminated by adding 100 μL of stop solution, and absorbance was measured at 450 nm within 10 min using a microplate reader (Infinite 200 PRO, Tecan, Switzerland). Standards at 10, 30, 90, 180, and 270 ng/mL were assayed under the same conditions. The limit of detection (LOD) and limit of quantification (LOQ) of the ELISA kit are 2.3 mg gliadin/kg food and 5 mg gliadin/kg food. Gluten content was calculated using RIDA^®^SOFT Win.NET (Z999, R-Biopharm AG, Darmstadt, Germany) based on gliadin content with the standard curve.

### 2.10. Molecular Docking

During food processing, starch can be hydrolyzed into dextrins and oligomeric fragments bearing reducing ends [36]. Such reducing carbohydrate fragments may react with epitope amino residues of allergenic proteins, modifying or masking IgE-binding epitopes, as reported for other allergen–carbohydrate systems [15,37]. Two representative allergenic wheat gluten subunits were selected as receptors from the UniProt database (https://www.uniprot.org/): α/β-gliadin A-IV (P02863), whose α-gliadin family harbors the immunodominant wheat T-cell epitopes [38], and HMW glutenin subunit Dx5 (P10388), classified among wheat gluten and allergen proteins [39]. Maltodextrin (CID 62698) was retrieved from PubChem (https://pubchem.ncbi.nlm.nih.gov) and docked against both proteins using AutoDock 4.2.6 with a 126 × 126 × 126 grid box. Short oligomeric starch fragments of rice and wheat (linear amylose and single-branched amylopectin) were constructed with a CHARMM-GUI Glycan Reader & Modeler [40]: rice amylose DP24 and amylopectin DP18 + DP6 (branched at residue 9), and wheat amylose DP28 and amylopectin DP20 + DP8 (branched at residue 10). The linear fragments fall within the DP 12–30 range of commercial maltodextrins [36], while the branch chain lengths correspond to typical amylopectin A-chain (DP 6–12) and B1-chain (DP 13–24) populations [41]. Pairwise docking of Dx5, α/β-gliadin A-IV, and the four starch fragments was then performed with HADDOCK3 [42]: 10,000 fully blind rigid-body conformations were embedded by UMAP based on contact fingerprints between protein amino acid residues and starch glucosyl residues and clustered via Ward’s linkage hierarchical clustering [43]. After scoring-based screening of the top 500 poses, the lowest-HADDOCK-score conformation was adopted for binding analysis. The pipeline was implemented in Python 3.10 and R 4.2.3, and structures were visualized and analyzed in open source PyMOL 3.10.

### 2.11. Statistical Analysis

All measurements were conducted on independent preparation batches (*n* = 3) and are expressed as mean ± standard deviation (SD). For gluten hydrolysates characterized before starch incorporation, for which no starch factor existed, one-way ANOVA followed by Tukey’s HSD test was used. Significance was set at *p* < 0.05. For the MRCPs, a two-way ANOVA was performed with gluten treatment (GO, GE, GC) and starch type (WS, RS) as fixed factors (Type III sum of squares). Residual normality and variance homogeneity were checked using the Shapiro–Wilk and Levene tests, respectively. When the treatment × starch interaction was significant, simple effects were examined, and estimated marginal means were compared within each starch (GO vs. GE vs. GC) and within each treatment (WS vs. RS) with Bonferroni adjustment. In the figures, lowercase letters (a–c) denote differences among GO/GE/GC within a starch, and uppercase letters (A–B) denote differences between WS and RS within a treatment. All statistical analyses and calculations were performed using IBM SPSS Statistics 27 (IBM Corp., Armonk, NY, USA) and OriginPro 2017C SR2b9.4.2.380 (OriginLab Corporation, Northampton, MA, USA).

## 3. Results and Discussion

### 3.1. Physicochemical and Functional Properties of Soluble Gluten Extracts

#### 3.1.1. Analysis of Mw Distribution 

**Figure 1:**
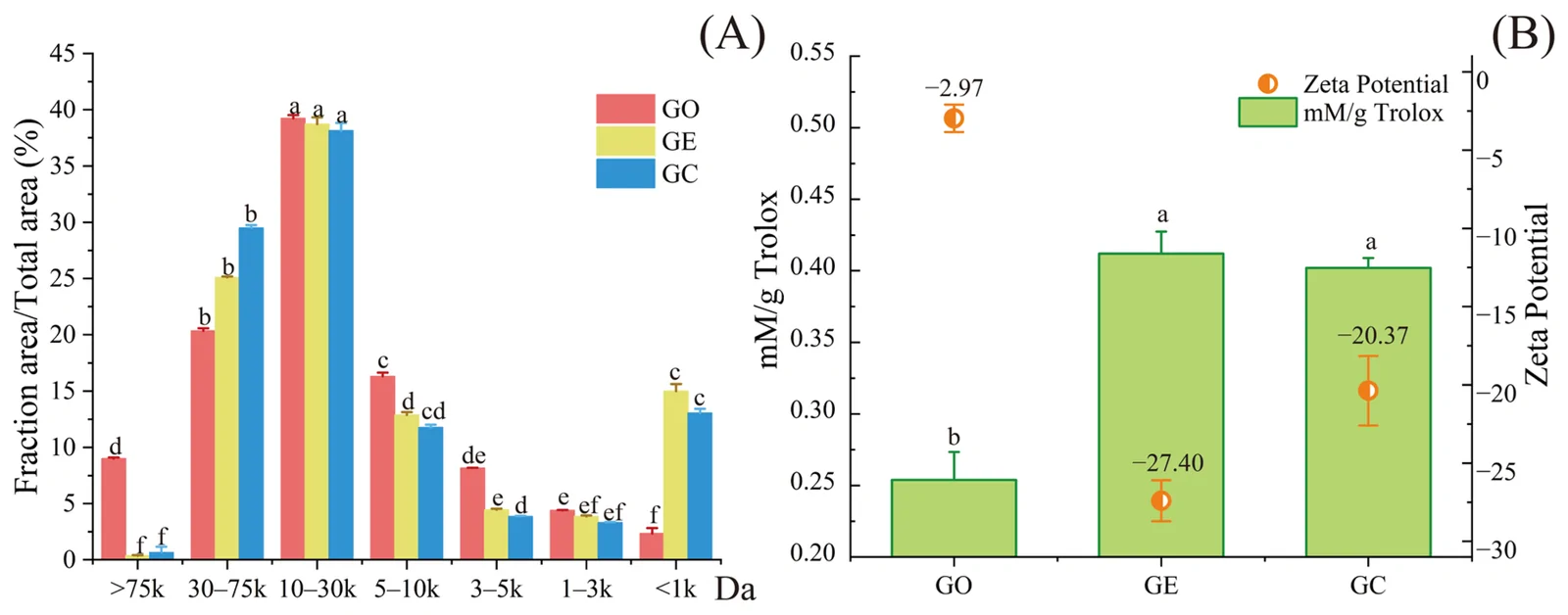
Properties and functions of gluten-processing-derived fractions. (**A**) The Mw distribution of soluble gluten hydrolysates analyzed by SEC, (**B**) ABTS·^+^ free radical scavenging effect and zeta potential of GO/GE/GC. a–f represent significant differences (*p* < 0.05) among molecular weight fractions in panel (**A**), and a–b represent significant differences (*p* < 0.05) among the GO, GE and GC samples in panel (**B**).

The Mw distribution of the soluble gluten extracts directly reflects the extent of hydrolysis (Figure 1A). In the GO group, species below 30 kDa dominated (10–30 kDa, 39.22%, <10 kDa, 31.21%), whereas all intact gluten protein types exceed 28 kDa (monomeric gliadins (28–55 kDa, ~50%), disulfide-linked HMW-gliadin oligomers (70–700 kDa, ~15%), and polymeric glutenins (0.7 to >10 MDa, ~35%)) [44,45]. The sub-30 kDa species of GO suggest hydrolytic peptides already present in the gluten preparation rather than intact subunits, while the >75 kDa fraction (9.08%) of GO corresponds to the solubilized portion of HMW-gliadin oligomers or HMW-GS x [44,45]. GE shifted mass down the Mw ladder: >75 kDa was almost cleared (9.08% → 0.34%), the 5–10, 3–5, and 1–3 kDa fractions decreased (16.31% → 12.88%, 8.17% → 4.44%, and 4.39% → 3.86%), and <1 kDa increased ~6.4-fold to 14.99%, identifying <1 kDa as the net sink of hydrolysis and explaining the GO > GE > GC order of the 5–10/3–5/1–3 kDa fractions. Unexpectedly, GC raised the 30–75 kDa fraction (25.08% → 29.50%) while slightly lowering all sub-10 kDa fractions compared with GE. Because SEC profiles represent relative proportions within the soluble phase, this pattern most likely reflects cavitation-mediated solubilization of previously insoluble aggregates that entered hydrolysis as subunit-sized intermediates faster than they could be converted to <1 kDa peptides, in line with the reported loosening of WG aggregates and facilitated enzyme attack by ultrasonic pretreatment [10,46]. Nevertheless, ~60–68% of soluble protein persisted in the 10–75 kDa range across treatments, implying incomplete hydrolysis and possible retention of immunoreactive sequences, attributable to the compact, intrachain S–S-stabilized and poorly accessible gliadin structures [45]. This indicated that ultrasound-assisted enzymolysis achieved an intermediate DH, which might endow GC with better interfacial and mechanical functionality than GE. In contrast, the immunoreactivity of the treated groups remains to be verified.

#### 3.1.2. Total Amino Acid (TAA) Composition

The TAA compositions and taste attributes of GO/GE/GC are given in Table 1 and Table 2. All samples were rich in Glx (Glu + Gln) and Pro but poor in Cys and Met. This matches gliadins, especially ω-gliadins, which are Gln/Pro-rich and almost devoid of sulfur AAs [44,45]. Given that proteolysis rapidly disperses gluten and glutenin aggregates into soluble, subunit-derived peptides [47], this composition suggests that the soluble fraction was enriched in gliadin-type (particularly ω-gliadin-like) subunits [45]. A previous study reported that Glu/Pro tend to rise first and then decline, whereas Cys/Met follow the reverse trend during proteolysis [47]. Here, Glx and Pro ranked GO > GE > GC, Cys GE > GC > GO, and Met GE > GO > GC. These shifts indicate that ultrasound altered the extent and cleavage pattern of the subsequent hydrolysis and thus the released AA profile [46]. Glutenin is cleaved faster than the compact, Pro/Gln-rich gliadin [47], while sonication loosens such compact structures and exposes more cleavage sites [46]. This change may further affect the specific immunoreactive residual sequences rich in Gln/Pro, which need to be further evaluated independently. The essential AA content (excluding Trp) ranked GC > GE > GO, indicating better essential AA retention in GC. Sweet/umami/sour AAs followed GO > GC > GE, while bitter and sulfurous AAs ranked GE > GC > GO, and mixed AAs GO > GE > GC. GE thus enriched bitter/hydrophobic species, whereas GC retained a profile close to GO at an intermediate peptide size. This agrees with peptide taste behavior in that large peptides mask hydrophobic residues, whereas small fragments expose hydrophobic, bitter termini [48]. These composition-based patterns suggest GC balanced an intermediate peptide size, retained essential AAs and even nutritional functions, and avoided the bitter-AA buildup seen in GE.

#### 3.1.3. Scavenging Effect of ABTS·^+^ and Zeta Potential

As presented in Figure 1B, GO exhibited the lowest antioxidant capacity, while GE showed marginally higher antioxidant capacity than that of GC. Ma et al. [52] demonstrated that EH and high acoustic energy disassembled β-lactoglobulin aggregates, yielding antioxidant peptides with more free radical-scavenging sites and smaller particle size. Hydrolysates enriched in low-to-medium-Mw fragments (~3000–500 Da) exhibited stronger antioxidant and radical-scavenging activities [10], whereas medium-Mw, hydrophobic gluten hydrolysates contribute markedly to ABTS·^+^ scavenging [20]. These results align with the higher hydrophobic AAs and lower-Mw fragments of GE/GC than counterparts of GO, which produce more functional abilities. Notably, GO had the least negative zeta potential, while GE showed more negative charge than GC. Gluten peptides exhibited negative potential at neutral pH due to carboxylic group ionization of Glu/Asp [53], and deamidation (converting Gln to Glu) enhanced this negative charge [49,53]. In this study, charge exposure may be caused by exposure of cleaved Glu/Asp-derived negative charge via EH. Nevertheless, the smaller negative charge, antioxidant activity, and low-Mw fraction in GC (vs. GE) suggest ultrasonication may induce new S–S formation or oligomerization, shielding AA side chains for EH [46,54]. This indicates ultrasonication mitigates excessive degradation of gluten’s higher-order structure caused by EH.

### 3.2. Browning Degree and Antioxidant Ability of Scavenging ABTS·^+^ for Mixtures

**Figure 2:**
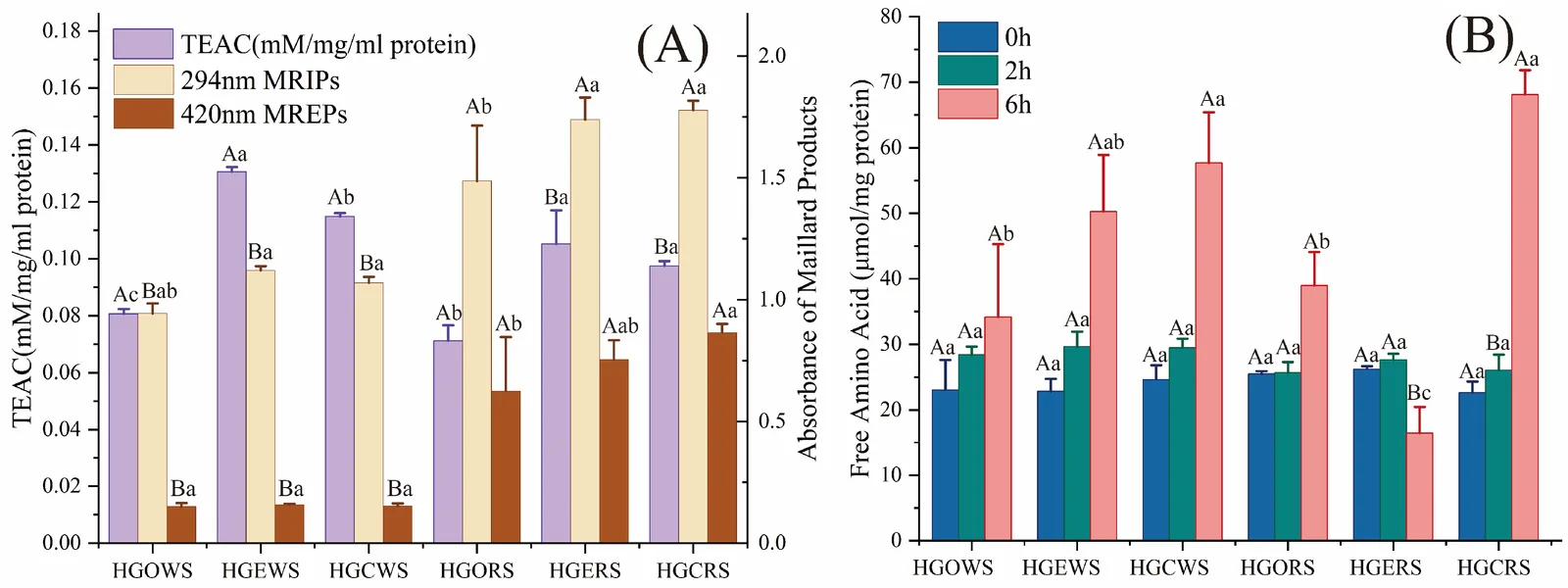
Properties and functions of MRCPs (denoted as HGOWS, HGEWS, HGCWS, HGORS, HGERS, HGCRS). (**A**) Browning degree and antioxidant ability of scavenging ABTS·^+^ for MRCPs; (**B**) analysis of AA contents in thermally treated protein–starch conjugates after simulated digestion at different time points. Note: For the final complexes, lowercase letters (a–c) indicate significant differences among GO, GE, and GC treatments under the same starch type, and uppercase letters (A–B) indicate significant differences between WS and RS starch under the same treatment. Heated ABTS-TEAC complexes (*n* = 3): Treatment and Starch *p* < 0.001, marginal non-significant interaction (*p* = 0.078). For A294 (*n* = 3), Treatment *p* = 0.005, Starch *p* < 0.001, interaction ns 0.415. For A420 (*n* = 3), Treatment ns 0.144, Starch *p* < 0.001, interaction ns 0.149.

Figure 2A shows the Maillard reaction extent (MRE) and ABTS·^+^ scavenging of the MRCPs. Each MRCP scavenged less ABTS·^+^ than its parent hydrolysate but followed the same ranking order as their hydrolysate counterparts. This likely reflects partial consumption of radical-scavenging small molecular peptides (SMPs) and free amino groups during microwave-driven MR, together with dilution by the starch phase [14,19]. GE/GC-derived composites nevertheless browned more and reached a higher MRE than the GO-derived ones, in line with their larger low-Mw peptide pool (the <1 kDa fraction presented in SEC detection). These SMPs are presumed to provide more free α-amino groups as MR sites [14,19]. In all samples, A294 (MRIPs) exceeded A420 (MREPs). A294 is commonly used as a proxy for colorless MR intermediates, which mainly comprise relatively stable Amadori/ketoa006Dine species that accumulate before late melanoidins [55]. Microwave heating has elsewhere been reported to raise A294/A420 yields relative to conventional heating through dielectric polarization [56]. On this basis, the A294-over-A420 pattern observed here would be consistent with a comparatively early, moderate stage of MR in most composites [19], which needs to be quantified directly thereafter. The WS–RS divergence is more complicated, involving gelatinization, reducing-sugar release, and peptide–sugar contact. WS and RS differ intrinsically in amylopectin content, granule size, gelatinization enthalpy, and resistant-starch fraction [57]. Across starch types, HxRS reached a higher MRE but lower ABTS·^+^ activity than HxWS (*p* < 0.05), and HGCX was marginally lower than HGEX. Smaller granules hydrate faster and offer a larger interfacial area [57]. During heat–moisture treatment, free α-amino groups at the gelatinized surface are consumed as melanoidins form and diffuse into the granules [58]. Limited microwave degradation may also form reducing-end dextrins or oligosaccharides [36,59]. It is postulated that the higher MRE of HxRS may suggest more frequent contact between low-Mw peptides and reducing-end dextrins at the faster-hydrating, larger RS interface. The higher ABTS·^+^ activity of HxWS could reflect greater retention of radical-scavenging moieties. The marginally lower activity of HGCX than HGEX is likewise consistent with shielding by higher-Mw peptides of GC. Overall, starch type appears to influence MRE and may shape the volatile profiles, while the exact roles of gelatinization and peptide–starch binding await direct measurement [57,58].

### 3.3. Assessment of the Digestibility of Gluten–Starch Complexes

As shown in Figure 2B, except for HGERS (which showed markedly decreased AAs at 6 h), AA release increased over time for all other complexes. All samples exhibited comparable AA release within the first 2 h, while the liberation of digestible AAs followed the ascending order HGCX > HGEX > HGOX at 6 h, with RS conjugates yielding greater AA release. EH combined with microwave heating promoted MR and gluten aggregation/cross-linking, which increased starch–protein interactions for masking modifications and reduced digestive sites, thus lowering total AAs during digestion [19,22]. Nevertheless, milk protein aggregates with elevated viscosity exhibited enhanced AA liberation, suggesting that AA release is governed by an interplay between protein aggregation status and enzymatic susceptibility during digestion [60]. For HGOXS, lower total AA release compared to treated complexes may be ascribed to intact gluten but not limited MRCPs formation, which mitigated digestive cleavage. Compared to HGCX, reduced AA release in HGEX may stem from increased MRCPs formation via higher SMP content during processing, which generates more covalent complexes and impairs proteolytic cleavage by digestive enzymes. HxRS displayed greater AA release relative to HxWS, perhaps owing to finer gelatinized RS rich in amylopectin, ultrasound-mediated gluten assembly [46], and lower SMP levels. The resultant loose aggregates and limited MR allowed easier protease access and hydrolytic breakdown of the complexes. The lowest AA release of HGERS may be caused by elevated compact, digestion-resistant aggregate formation because of more gluten SMPs coupled with higher heating-induced starch gelatinization/MRCPs formation [15]. Additionally, encapsulation of SMPs or AAs by gelatinized starch may further hinder the detection of AAs in supernatants. However, these findings still require further validation.

### 3.4. Analysis of Color Properties

**Figure 3:**
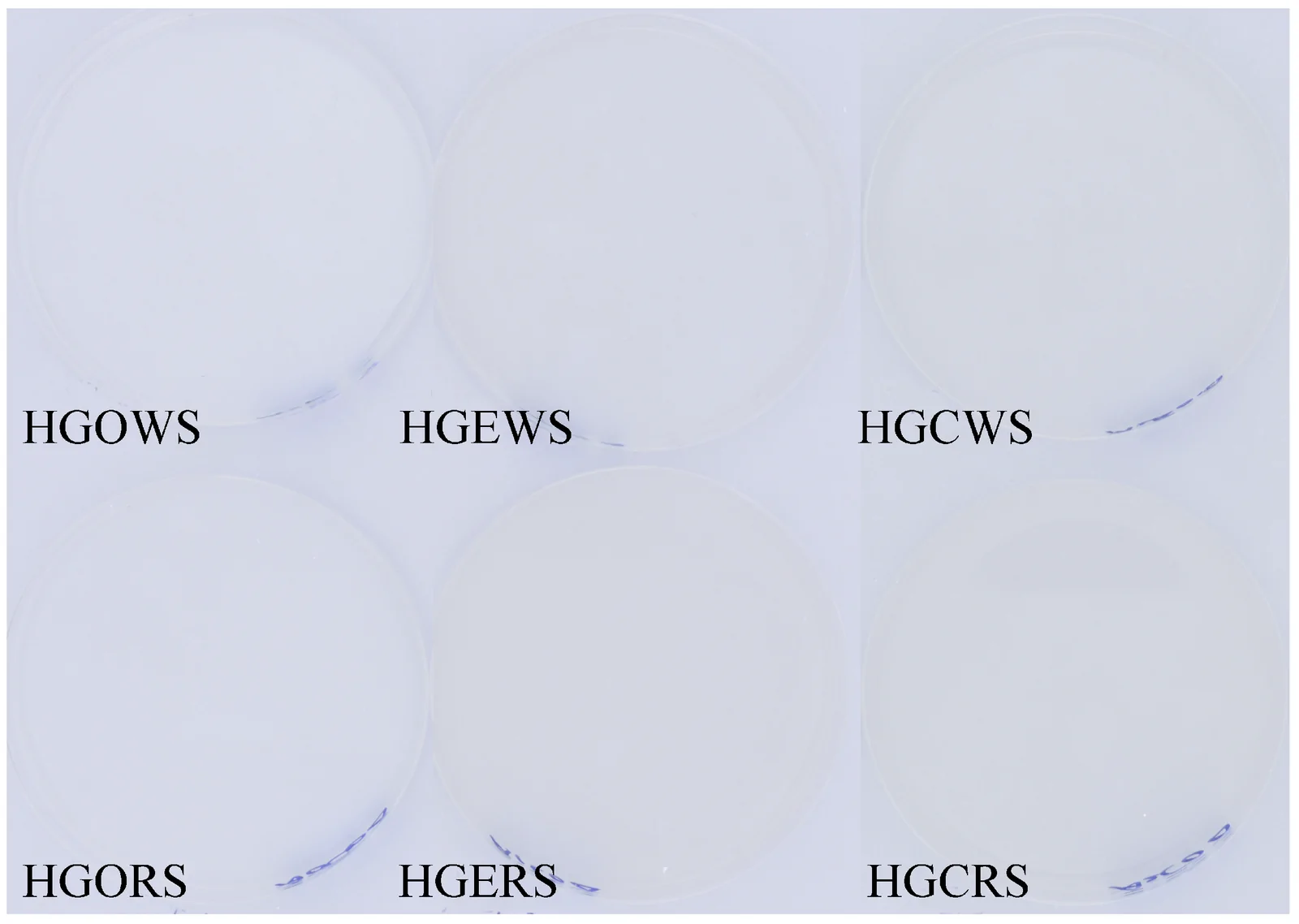
The electronic eye colorimetry of gluten-derived MRCPs in aqueous solution.

As shown in Supplementary Figure S1, HGEX appeared a relatively darker yellowish color than other samples. The color of HGCX was the most uniform, and HxRS composites exhibited a darker color than the corresponding HxWS composites. Color attributes of all composites were accurately quantified as shown in Figure 3 and Table 3. L* decreased in the order HGOX > HGEX > HGCX, with HxWS conjugates exhibiting higher L* values than HxRS counterparts. Maillard-derived pigments lower L* [14], whereas larger aggregates formed during heating can scatter/reflect light and partly offset darkening [61]. The highest L* in HGOX is thus consistent with its lower MRE, while the intermediate L* in HGEX may reflect a balance between pigment formation and light scattering by larger MR conjugates. HGEX showed the highest positive a* value, while the negative a* of other samples were displayed as HGCWS < HGOWS < HGEWS and HGORS < HGCRS < HGERS. The link between melanoidins and a* is not straightforward [62]; the more positive a* of HGEX is nonetheless consistent with its higher MRIPs (Figure 2). All negative b* values are present in the order HGOWS < HGCWS < HGEWS and HGCRS < HGERS < HGORS. A higher resistant-starch fraction darkens baked matrices [63], while advanced MR shifts b* toward more negative values [30]. This explains the higher MRE in HGE(C)RS vs. HGE(C)WS (Figure 2). A higher resistant-starch fraction lowers b* and darkens baked matrices [63], while MR shifts b* toward more negative (bluer) values [30]; accordingly, HGE(C)RS was bluer than HGE(C)WS counterparts. C* followed the same trend as b*. HGEWS had the highest C* in HxWS, while HGORS peaked in HxRS. The C* of the heated glucose–ammonium system increased first, then decreased at 100 °C and continuously decreased at 110 °C or 120 °C [30]. A study also showed that C* increased in ultrasound-treated ultrafiltrated smoothhound viscera protein-sucrose conjugates [64]. For HxWS, C* variation was consistent with the browning degree of MRIPs, confirming that Maillard pigment formation contributed to the color of MRIPs. For HxRS, the value order of C* was contrary to the browning degree, and HGORS showed the highest C*. One tentative explanation is easier gelatinization of the smaller RS granules, which would let low-Mw GE/GC chromophores diffuse into the granule more readily than the high-Mw GO material [58]. W* followed HGEX < HGCX < HGOX and was lower in HxRS than HxWS, trending with MRE as reported for heated protein–sugar conjugates [64]. ΔE* showed the same ordering and was highest in HGEX, although these differences were not always significant (*p* > 0.05); the relatively high ΔE* of HGOX may instead reflect light scattering by its larger aggregates [61], in which microwaving promoted aggregate formation [19]. The lowest ΔE values of HGCX also demonstrate more homogeneous color distribution throughout the matrix, which suggests HGCX is suitable for subsequent food product development.

### 3.5. Volatile Flavor Components Determination

The profiles of volatile flavor components (with RI) for the different composites and their corresponding descriptions are shown in Table 4. A higher relevance index reflects a better chromatography matching degree of detected peaks with the standard library. All composites contained methanethiol (burnt, sulfur notes), a volatile commonly derived from the degradation of sulfur-containing amino acids in thermally processed starch–protein systems [33]. For HxRS, higher EH intensified the MR during microwave treatment: HGERS developed prominent creamy roasted notes and HGCRS the most native wheat-like fresh grassy and nutty malt aroma, whereas HGORS, with the least MRE, retained only minimal roasted wheat notes. Within HxWS, HGOWS was dominated by strong smoky popcorn notes despite its lower MRE, suggesting a pathway beyond MR, since phenolic acids retained in the matrix can be thermally converted into smoky phenolic volatiles during heating [32,65], while popcorn-like notes are typical MR-derived odorants [33]. HGEWS showed the richest balanced flavor profile, and HGCWS the strongest raw-material character with pure fresh wheat and spicy notes. This contrast between HxRS and HxWS thus reflects the richer endogenous flavor precursors (reducing sugars and phenolic acids) in WS, which sustain both MR and phenolic-acid conversion, whereas RS, being more highly refined, lacks such precursors, as phenolic compounds are mainly located in the outer layers of cereal grains and largely removed during refining [66], as also reflected by the weaker roasty, popcorn-like aroma reported for rice compared with wheat systems [32].

**Figure 4:**
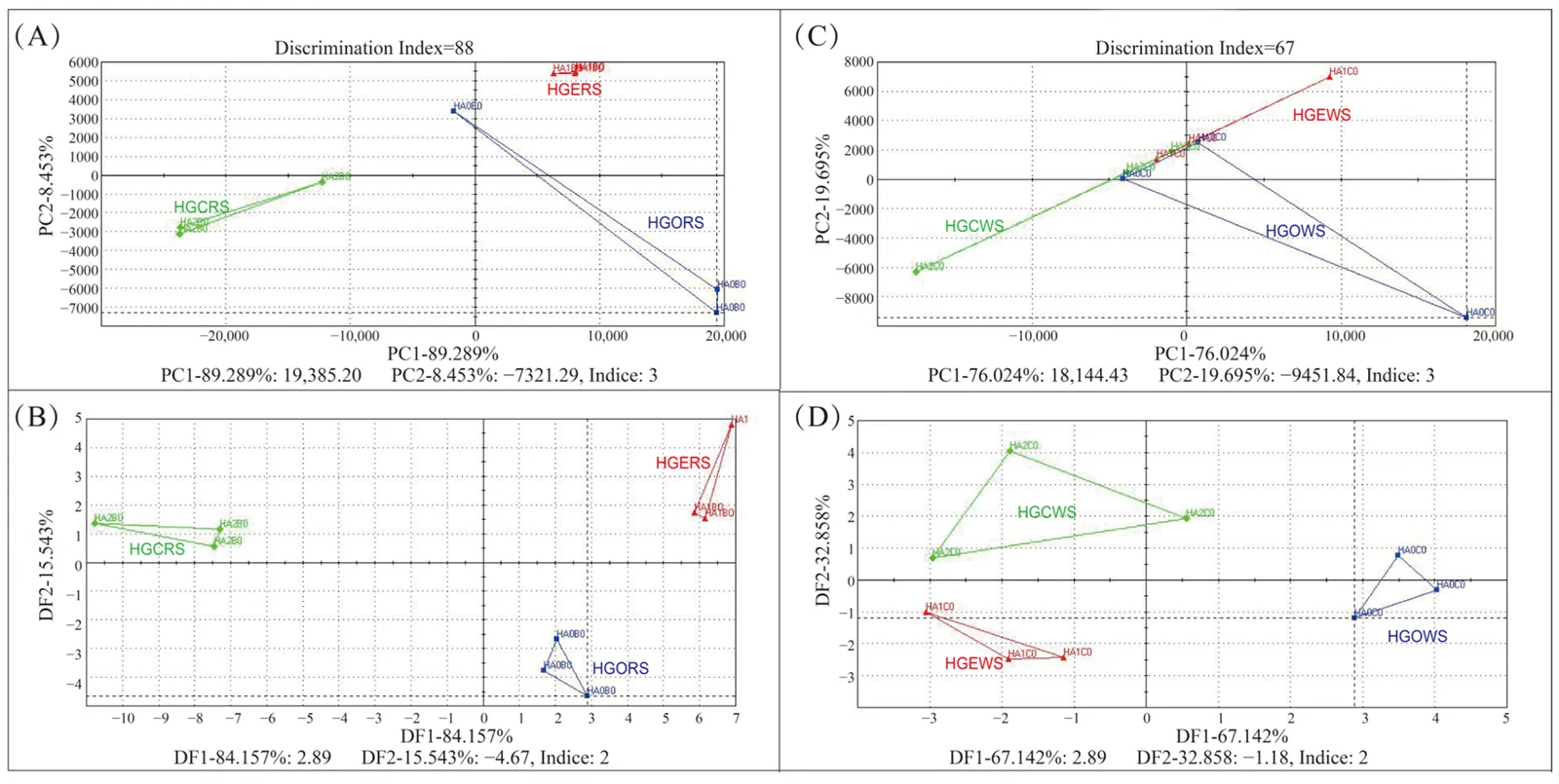
Principal components analysis and discriminant function analysis of starch–protein Maillard complexes. (**A**) and (**B**) represent the PCA and DFA for HxRS conjugates; (**C**) and (**D**) represent the PCA and DFA for HxWS conjugates. (“■” represents HGOWS or HGORS, “♦” represents HGEWS or HGERS, “▲” represents HGCWS or HGCRS).

PCA and DFA were applied to the auto-scaled volatile fingerprints (Figure 4; Table 5). The first two PCs captured 95.719% (HxWS) and 97.742% (HxRS) of the total variance, while the two discriminant factors together accounted for 100% of the between-group variance. PCA gave close or partly overlapping clusters, with clearer separation in HxRS and the widest replicate spread for HGOX. Along PC1, the sequentially treated HGCX was characterized by markers of native cereal aroma: the Strecker aldehyde 3-methylbutanal [32], hexanal derived from lipid oxidation, and methyl eugenol. Along PC2, HGEX shifted toward Maillard-derived sulfur volatiles (bis(2-methyl-3-furanyl) disulfide, 2-methylthiophene) generated by the thermal degradation of cysteine [33], whereas HGORS/HGCRS lay on the opposite side of the axis and were devoid of such advanced Maillard products. In HxWS, HGOWS was additionally associated with guaiacol, a smoky phenolic marker consistent with the endogenous phenolics concentrated in the germ and bran of the wheat matrix [66], together with two further cysteine-derived volatiles, 2-acetylthiazoline and dimethyl trisulfide [33].

DFA sharpened this separation with wider cluster spacing, especially for HxRS, in line with the higher discriminating power of DFA over PCA reported for aroma fingerprinting [34,35]. For HxRS, DF1 (84.16%) separated HGCRS from HGORS/HGERS using the same aldehyde markers as PC1, whereas DF2 (15.54%) distinguished HGORS by hexyl heptanoate and trans-4,5-epoxy-(E)-2-decenal as negative markers; the latter is a metallic lipid-oxidation product, so that HGORS was defined by lipid- and ester-derived volatiles rather than by thermally generated ones, complementing its position opposite HGEX along PC2. For HxWS, DF1 (67.14%) separated HGEWS from HGCWS and DF2 (32.86%) isolated HGCWS. Beyond the qualitative grouping, the relative aroma index (RAI) [34] ranked the treatment contrasts as HGEX–HGCX > HGOX–HGCX > HGOX–HGEX and was higher within HxWS than within HxRS (Table 5). This matrix difference may in turn reflect the richer pool of endogenous phenolics concentrated in the germ and bran of wheat [66], together with lipid precursors generating additional aldehydic volatiles. The interpretation remains tentative, however, because precursor abundance was not directly quantified. Both methods thus converged on the same layered pattern: HGCX retained a native cereal profile through mild Strecker degradation and lipid oxidation, HGOX/HGEX developed stronger thermally generated notes dominated by cysteine-derived sulfur volatiles, and the rice-starch matrix allowed the clearest treatment separation.

### 3.6. ELISA Assays for R5-Reactive Gluten

R5-reactive gluten decreased stepwise across treatments (Table 6). HGOX remained above 20 ppm, HGEX fell to around 15 ppm, and HGCX fell to 8.31–9.82 ppm, i.e., below 10 ppm and the 20 mg/kg Codex Alimentarius threshold [67]. The ordered combination thus lowered detectable R5-immunoreactive gluten more than enzymatic hydrolysis alone. R5 is known to recognize the Q/P-rich gliadin motifs QQPFP, QQQFP and LQPFP [25]. It has been reported that ultrasonic pretreatment can loosen compact protein aggregates and expose additional cleavage sites, facilitating later enzymatic fragmentation of epitope-bearing sequences [24]. In our samples, the larger low-Mw peptide pool in GC is consistent with this route and may have contributed to its lower R5 signal. Thermal treatment alone cannot abolish immunoreactivity, as it mainly alters conformational epitopes while linear Q/P motifs persist [68], which is in line with the high residual signal of HGOX. Previous studies further indicated that glycation and food ingredient interactions (e.g., starch) during heating can mask antibody epitopes and lower immunochemical detection [15,68]. Accordingly, conjugation of peptide amino groups with reducing-end dextrins during microwaving may have partly shielded residual motifs in HGCX. Within this analytical scope, enzymatic cleavage and possible glycation-mediated shielding may have operated together. The data suggest that ordered multi-step processing may provide a basis for developing reduced-gluten ingredients. However, the individual contributions of both factors, and orthogonal product-level verification with complementary antibodies (e.g., G12) and mass spectrometry, still need to be carried out.

### 3.7. Interaction Between Wheat Gluten Subunits and Starch Fragments

**Figure 5:**
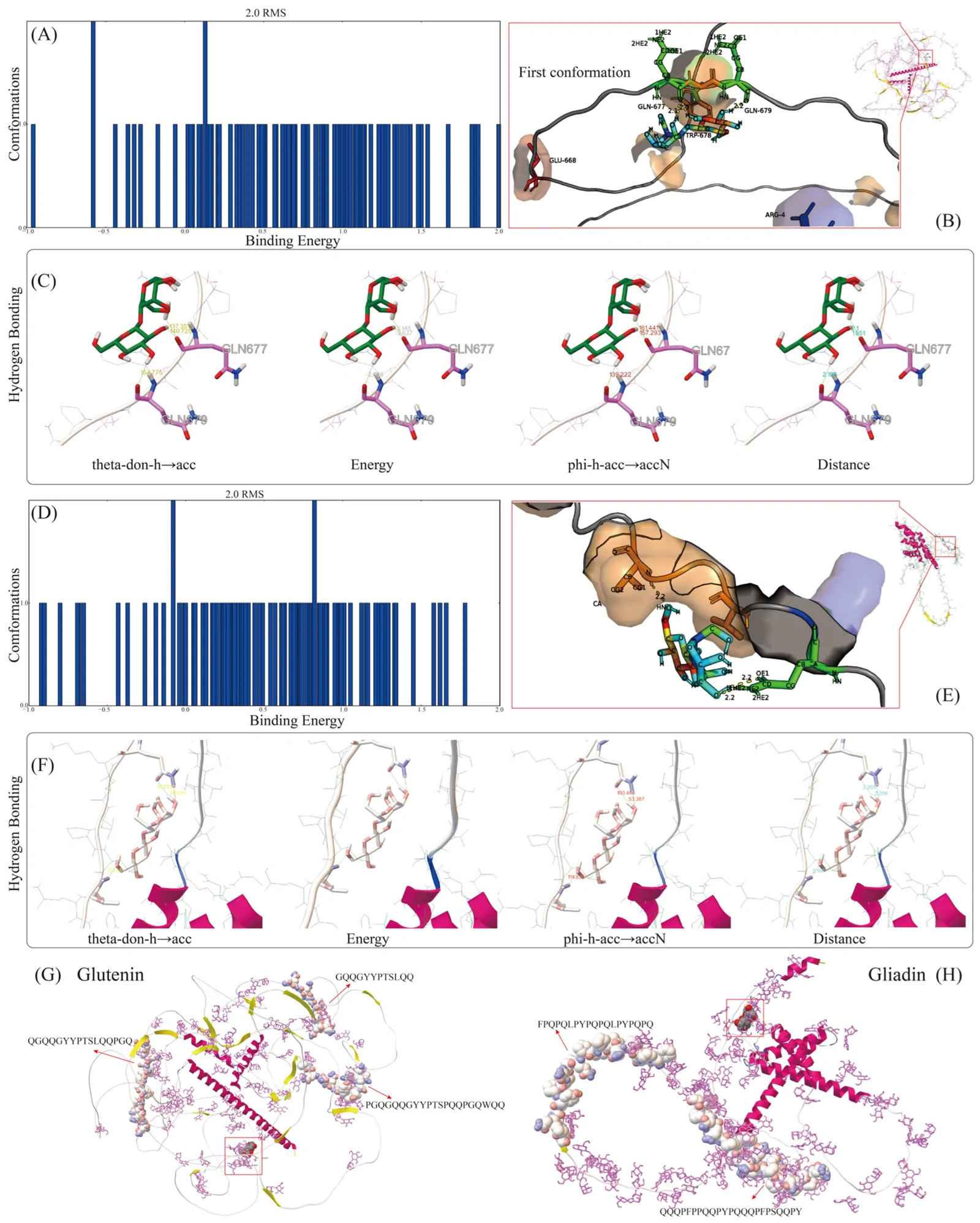
Molecular interaction between starch maltodextrin (ligand) and gluten subunits (receptor). (**A**) Cluster of all 100 conformations of ligand with glutenin, (**B**) magnified details of all the interaction between first lowest energy conformation between starch maltodextrin and glutenin (structure without red-box highlighting is the intact composite), (**C**) the detailed hydrogen bond interaction between first lowest energy conformation of starch maltodextrin and glutenin (don: hydrogen bond donor, acc: hydrogen bond acceptor, accN: heavy atoms connected to acceptor atoms), (**D**) cluster of all 100 conformations of ligand with gliadin, (**E**) magnified details of all the interaction between first lowest energy conformation of between starch maltodextrin and gliadin (structure without red-box highlighting is the intact composite), (**F**) the detailed hydrogen bond interaction between first lowest energy conformation of starch maltodextrin and gliadin, (**G**) all 100 conformations of ligand with glutenin (the epitopes shown in CPK, the red circle means the first conformation of the ligand), (**H**) all 100 conformations of ligand with gliadin (the epitopes shown in CPK, the red circle means the first conformation of the ligand).

Figure 5 illustrates the AutoDock-predicted interaction of glutenin/gliadin with maltodextrin. Binding energies (BEs) were −0.96 and −0.92 kcal/mol, with inhibition constants (Ki) of 196.29 and 211.84 mM for glutenin and gliadin, respectively. Total interaction energy was −4.39 and −4.34 kcal/mol for glutenin and gliadin. The weakly negative values indicate thermodynamically favorable but low-affinity binding, mediated predominantly by hydrogen bonds involving Gln/Glu residues together with hydrophobic contacts. These binding modes between maltodextrin and gluten subunits raise the hypothesis that bound maltodextrin may sterically shield epitope-rich regions.

**Figure 6:**
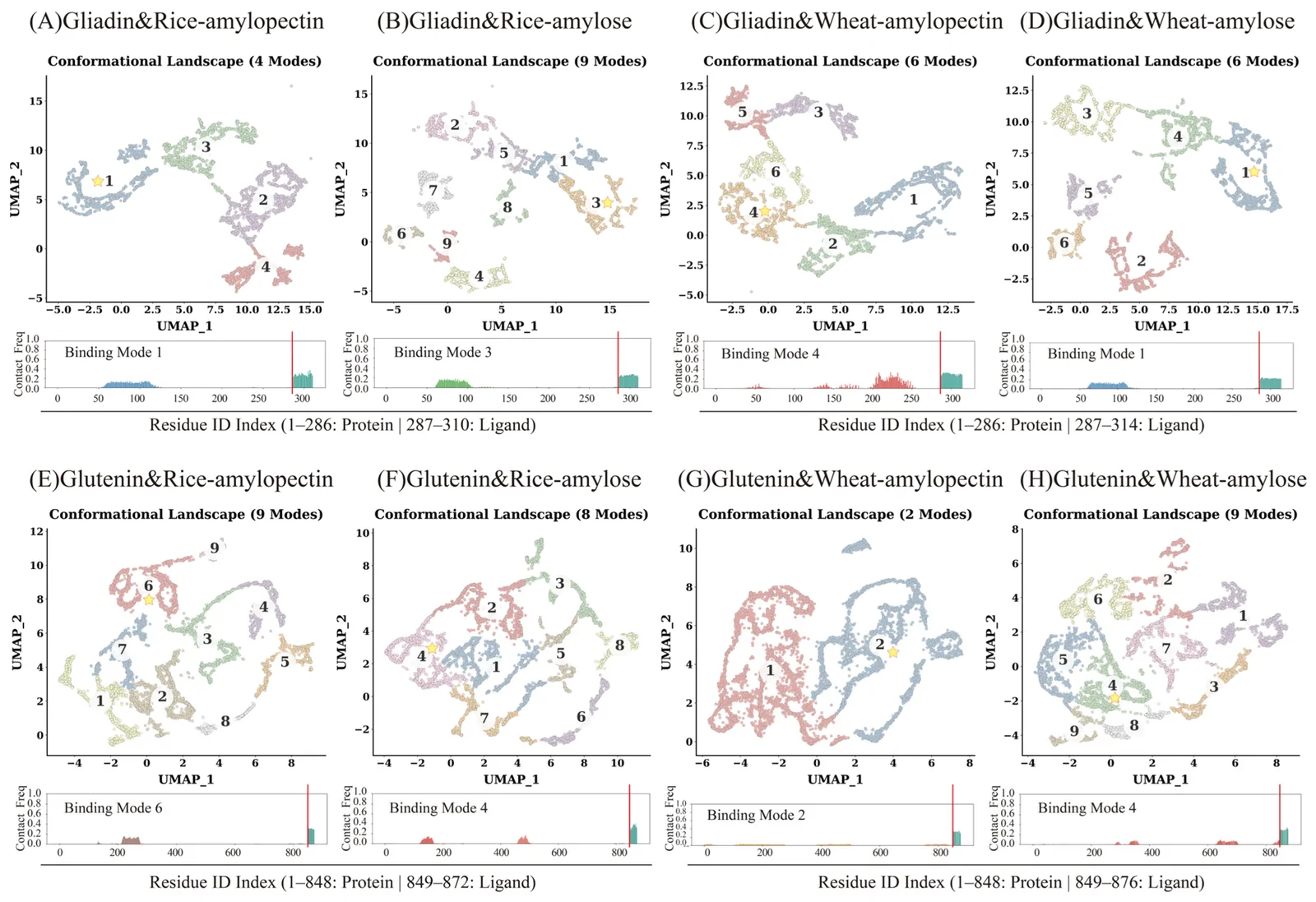
UMAP projections of 10,000 rigid-body conformations of protein–starch Maillard complex landscapes based on residue binding patterns, colored by amino acid–glucose residue interface contact fingerprints (residue contact threshold < 5.0 Å; the solid red line marks the boundary between the protein sequence and the starch fragment sequence). (**A**) Gliadin and rice amylopectin, (**B**) gliadin and rice amylose, (**C**) gliadin and wheat amylose, (**D**) gliadin and wheat amylopectin, (**E**) glutenin and rice amylopectin, (**F**) glutenin and rice amylose, (**G**) glutenin and wheat amylose, (**H**) glutenin and wheat amylopectin.

Average contact frequencies for each residue between protein subunits and starch fragments were compared to identify different binding patterns and the best binding modes, as shown in Figure 6 (individual dots stand for unique docking poses) and Table 7. Rigid-body docking revealed richer binding modes for RS fragments interacting with gluten subunits, while wheat amylopectin exhibited only two binding patterns toward glutenin. The abundant interaction sites of RS fragments increase reactive group contact probability, potentially enhancing the MRE of modified gluten–RS systems. Studies reported that Gln would be deamidated to Glu by transglutaminase 2 (TG2) in vivo, which further activates epitopes and stimulates T cells [38,69]. The major IgE-binding epitopes of HMW glutenins are QQPGQ, QQPGQGQQ, and QQSGQGQ [70], while glutenin-derived T cell epitopes such as QGYYPTSPQQS drive the gluten-specific mucosal T cell response [71]. For gliadins, the primary IgE epitopes of ω5-gliadin share the consensus sequence QQX_1_PX_2_QQ (X_1_ = L, F, S, or I, X_2_ = Q, E, or G) [7], and IgE-binding regions of α-gliadin include ^53^QPYLQQFPQQPFPPQLP^72^ and ^77^QSFPPQQPYPQQ^88^ [72]. These Gln-rich epitopes underlie urticaria, WDEIA, and atopic eczema dermatitis syndrome [70,72]. Optimal binding conformations showed that RS fragments preferentially target the 60–115 residue region of the docking construct. This region falls within this epitope-rich repetitive domain and harbors multiple QQPYP/PQQPYP motifs (e.g., ^61^QQPYP^64^ and ^97^QQPYP^101^), the repetitive units of α/β-gliadins derived from the ancestral QQPQPFP motif [8]. These motifs overlap with characterized T-cell epitopes such as DQ2.5-glia-α4 (PQPEQPYPQ) [38]. On the other hand, WS fragments conservatively bind the non-epitopic 826–848 region of glutenin in silico. Thermodynamically, rice amylopectin forms more stable complexes with both gliadin and glutenin than wheat amylopectin, while amylose exhibits the opposite trend, suggesting that branched structural features may be a major contributor to starch–gluten binding stability in silico.

**Figure 7:**
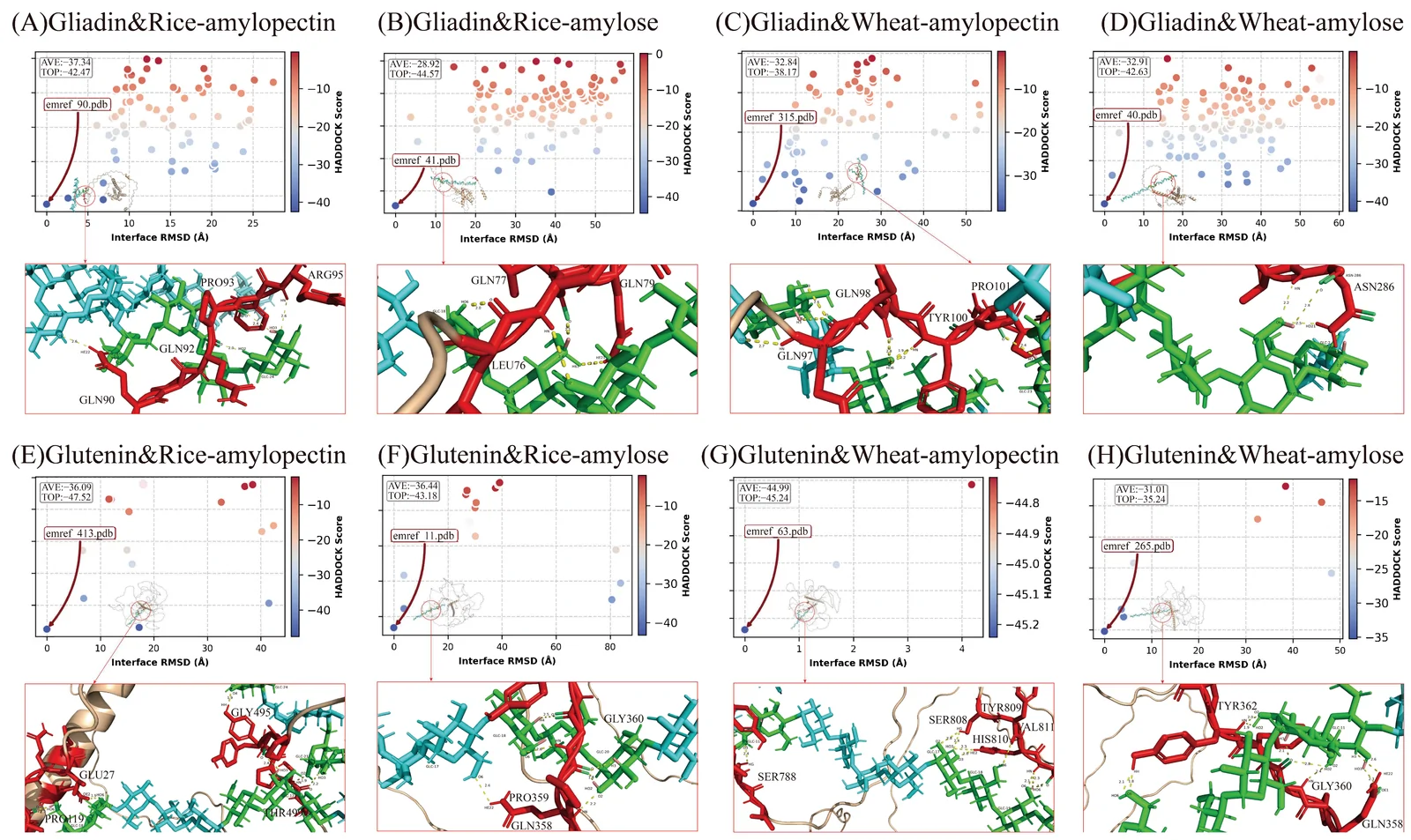
Distribution of HADDOCK docking results and identification of the optimal conformation. (**A**) Gliadin and rice amylopectin, (**B**) gliadin and rice amylose, (**C**) gliadin and wheat amylose, (**D**) gliadin and wheat amylopectin, (**E**) glutenin and rice amylopectin, (**F**) glutenin and rice amylose, (**G**) glutenin and wheat amylose, (**H**) glutenin and wheat amylopectin. (wheat: protein subunits, red: AAs that form H-bonds within protein subunits, blue: starch fragments, green: glucose that forms hydrogen bonds within starch fragments).

Figure 7 illustrates the structural landscapes, quality assessments, and molecular conformations, following flexible refinement and energy minimization of screened rigid-body. BEs and intermolecular forces for the various protein–starch fragment complexes were detailed in Table 7 and Table S2. Further flexref, emref, and mdref refinements and conformational energy landscape analysis confirmed that starch fragments exhibit broad-spectrum, multi-pocket, and multi-stable binding toward gliadin. Starch–gliadin docking was dominated by van der Waals/hydrophobic stacking supplemented by Gln/Pro hydrogen bonds. The rice-amylopectin–gliadin model occupied the lowest-energy conformations with the largest buried surface (emref −44.6; BSA 749.9; vdW −37.1; total −55.8). Whereas gliadin–amylopectin hydrogen-bond numbers were equal for rice and wheat (4 vs. 4), its hydrogen-bond donors clustered in the Gln/Pro-rich repetitive segment (Gln65–Gln98, Pro93) that overlaps R5-type QQPFP immunodominant regions [25]. Starch–glutenin binding was instead governed mainly by electrostatics, with more hydrogen bonds for rice (9 vs. 6). But near-zero desolvation only exists in the rice models and fewer dominant stable conformations, indicating more transient, lower-probability contacts (weakest for wheat-amylose: BSA 304.4; total −41.9). At the computational level, these in silico modes suggest broad burial of gliadin epitope-rich surfaces by rice amylopectin rather than single-site blocking, alongside transient electrostatic contacts with glutenin. This tendency is partly reflected by R5-ELISA, where treatment dominated the gluten content (ηp^2^ = 0.979, *p* < 0.001) while starch type was non-significant (*p* = 0.055), with RS lower than WS only in GO and no starch difference in GE/GC. The docking results thus offer a putative basis for possible changes in epitope accessibility. They also provide a putative molecular basis for the improved overall quality of modified gluten hydrolysate–RS complexes and lend molecular-level support to the combined modification potential of sequential processing. However, further experimental validation (e.g., multiple epitope peptide competition assays) is warranted.

## 4. Conclusions

Modified gluten hydrolysates were prepared by ultrasonic pretreatment followed by enzymatic hydrolysis, blended with WS or RS, and microwave-heated to establish a simplified gluten hydrolysate–starch composite model (MRCPs). The sequentially treated HGCX composites showed comprehensively improved functional properties with enhanced ABTS·^+^ scavenging activity, increased release of ninhydrin-reactive amino groups after in vitro digestion, favorable instrumental color and volatile profiles (mild wheat-like and Maillard-type notes), and the lowest residual R5-reactive gluten (8.31–9.82 ppm). These improvements were associated with an enriched AAs composition, increasing SMPs (>1 kDa), and controlled formation of MR products. According to the docking models, putative starch fragment–gluten subunit interactions in which there were Glu/Gln–glucose hydrogen bonding and hydrophobic contacts provide broad, multi-site surface coverage near immunodominant sequences (e.g., ^61^QQPYP^64^, ^97^QQPYP^101^, ^78^LQPFP^82^). These computational patterns are consistent with spatial overlap of both linear and putative conformational epitope regions in the modeled complexes. Overall, the sequential combination of ultrasound-assisted enzymatic hydrolysis, starch incorporation, and microwave heating improved the color, volatile profile, and functionality, and lowered the R5-immunoreactivity of gluten hydrolysate–starch composites at the laboratory scale. This work offers a process-oriented reference for optimizing the comprehensive quality of gluten-containing ingredient systems.

## Figures and Tables

**Table 1:** The AA tastes and compositions of gluten hydrolysates.

% (Taste)	GO	GE	GC	Gluten [49]
Cys (sulfurous/bitter)	0.81	1.63	1.34	2.23
Met (sulfurous/bitter)	0.09	0.48	0.04	1.02
Phe (bitter)	4.83	4.82	4.76	3.70
Ile (bitter)	2.38	2.71	2.70	3.38
Leu (bitter/flat)	5.30	5.21	5.27	6.16
Val (bitter/slightly sweet)	3.20	3.36	3.33	4.05
Pro (sweet/slightly bitter)	13.57	13.56	13.04	18.8
Gly (sweet)	3.97	3.62	3.82	5.89
Ala (sweet)	2.45	2.19	2.29	3.81
Tyr (bitter)	2.85	3.01	3.12	2.07
Thr (sweet)	2.49	2.11	2.34	3.06
Ser (sweet)	4.37	4.11	4.47	5.72
His (bitter)	3.55	4.44	3.79	4.29
Arg (bitter)	3.26	3.54	3.96	3.62
Lys (bitter/sweet)	1.46	1.19	1.49	1.98
Glx(Glu + Gln) (umami/sour/slightly sweet)	42.59	41.75	41.31	26.2
Asx(Asp + Asn) (umami/sour/slightly sweet or slightly bitter)	2.81	2.28	2.93	3.37

Note: The first-appearing flavor is the dominant flavor reported in most of the literature [50,51].

**Table 2:** The sum of AA tastes of gluten hydrolysates based on AA compositions.

Taste (%) [50,51]	GO	GE	GC
Sulfurous(Cys + Met)	0.90	2.11	1.38
Sweet(Gly + Ala + Thr + Ser)	13.28	12.03	12.92
Sour(Glx(Glu + Gln) + Asx(Asp + Asn))	45.40	44.03	44.24
Umami(Glx(Glu + Gln) + Asx(Asp + Asn))	45.40	44.03	44.24
Bitter(Phe + Ile + Leu + Tyr + His + Arg)	22.17	23.73	23.60
Mixed and complex flavor(Val + Pro + Lys)	18.23	18.11	17.86

**Table 3:** The electronic eye colorimetry of gluten-derived MRCPs.

Sample	L*	a*	b*	C*	W*	ΔE*
HGOWS	96.90 ± 0.08 ^Aa^	−0.50 ± 0.14 ^Aa^	−5.35 ± 0.15 ^Aa^	5.37 ± 0.13 ^Bb^	80.76 ± 0.96 ^Aa^	0.93 ± 0.64 ^Aa^
HGORS	96.05 ± 0.37 ^Ba^	−0.57 ± 0.04 ^Ab^	−6.81 ± 0.64 ^Bb^	6.83 ± 0.64 ^Aa^	68.70 ± 5.84 ^Bb^	0.32 ± 0.30 ^Aa^
HGEWS	95.23 ± 0.06 ^Ab^	0.22 ± 0.01 ^Ab^	−7.00 ± 0.41 ^Ab^	7.01 ± 0.41 ^Aa^	64.06 ± 3.14 ^Ab^	1.01 ± 0.69 ^Aa^
HGERS	94.54 ± 0.21 ^Ab^	0.32 ± 0.00 ^Aa^	−6.24 ± 0.13 ^Aab^	6.24 ± 0.14 ^Aab^	65.56 ± 0.28 ^Aa^	0.63 ± 0.08 ^Aa^
HGCWS	94.93 ± 0.56 ^Ab^	−0.56 ± 0.01 ^Ab^	−6.09 ± 0.00 ^Aab^	6.11 ± 0.00 ^Aab^	68.35 ± 2.83 ^Ab^	0.67 ± 0.28 ^Aa^
HGCRS	94.36 ± 0.03 ^Ab^	−0.44 ± 0.01 ^Ab^	−5.70 ± 0.11 ^Aa^	5.72 ± 0.11 ^Ab^	67.78 ± 0.76 ^Aa^	0.25 ± 0.24 ^Aa^

Note: For the final complexes, lowercase letters (a–b) indicate significant differences among GO, GE, and GC treatments under the same starch type, and uppercase letters (A–B) indicate significant differences between WS and RS starch under the same treatment. Lightness (L: 0 = black, 100 = white, treatment F(2,6) = 46.91, *p* < 0.001, starch F(1,6) = 17.65, *p* = 0.006), red–green value (a: +a = red, −a = green, treatment F = 226.30, *p* < 0.001), yellow–blue value (b: +b = yellow, −b = blue, interaction F = 13.72, *p* = 0.006), whiteness (W*, interaction F = 5.99, *p* = 0.037), chroma (C*, interaction F = 13.78, *p* = 0.006), and color difference (ΔE*, ΔE* did not differ among groups (all *p* > 0.05)).

**Table 4:** Detected volatile compounds, relative index and flavor-related descriptors of MRCPs.

Sample	Odor Description	Major Compound Name (Relevance Index)
HGOWS	Smoky roasted flavor	2-Acetylthiazoline (74.30), Dimethyl trisulfide (44.29), Guaiacol (95.52), Indole (38.26), Methanethiol (47.34), Methional (73.62), Methyl cinnamate (32.56), 2,3-Pentanedione (89.35)
HGEWS	Fresh wheat notes, creamy sweet notes, light roasted notes and fermented undertone	Beta-ionone (45.62), Bis(2-methyl-3-furanyl)disulfide (69.35), Hexanal (92.25), Indole (70.79), Methanethiol (42.16), 2-Methylthiophene (74.68), Myristicin (86.80), 2,3-Pentanedione (96.01)
HGCWS	Pure and full spicy wheat aroma, close to native wheat flavor	Hexanal (93.99), Indole (56.45), Isoeugenol (85.64), Methanethiol (47.50), 2-Methyl-2-propanol (67.09), 3-Methylbutanal (74.46), Myristicin (83.10), Skatole (65.07)
HGORS	Minimal and pure basic roasted cereal aroma	Hexyl heptanoate (32.96), Indole (38.26), Methanethiol (46.23), Myristicin (81.17), Trans-4,5-epoxy-(E)-2-decenal (30.25)
HGERS	Full and rich creamy & roasted complex flavor, sufficient Maillard aroma	Bis(2-methyl-3-furanyl) disulfide (75.54), Hexanal (90.15), Indole (50.05), Isoeugenol (51.84), Methanethiol (45.91), 3-Methylbutanal (64.68), 2-Methylthiophene (77.58), Myristicin (84.97), 2,3-Pentanedione (97.09), Skatole (38.24)
HGCRS	Fresh and pure raw cereal wheat aroma, close to native wheat protein flavor	Hexanal (94.62), Indole (48.59), Methanethiol (46.72), Methyl eugenol (64.11), Myristicin (85.89), 2-Methyl-2-propanol (65.40), 3-Methylbutanal (76.62), Trans-4,5-epoxy-(E)-2-decenal (30.01)

**Table 5:** Diversity analysis and comparison of various MRCPs.

Product Names	Reference Samples	Distances	Percent Value	Pattern Discrimination Index (%)
HGOWS	HGEWS	10,248.78	0.00	28.23
HGOWS	HGCWS	32,328.65	0.00	76.13
HGEWS	HGCWS	28,362.75	0.00	90.88
HGORS	HGERS	6837.78	0.00	13.29
HGORS	HGCRS	12,989.53	0.00	31.2
HGERS	HGCRS	11,301.58	0.00	39.48

**Table 6:** R5-immunoreactive gluten content of various MRCPs.

Sample	Gluten Content ppm (mg/kg)
HGOWS	28.32 ± 1.24 ^Aa^
HGORS	25.11 ± 0.81 ^Ba^
HGEWS	16.56 ± 2.48 ^Ab^
HGERS	14.35 ± 0.82 ^Ab^
HGCWS	8.31 ± 0.63 ^Ac^
HGCRS	9.82 ± 0.85 ^Ac^

Note: For the final complexes, lowercase letters (a–c) indicate significant differences among GO, GE, and GC treatments under the same starch type, and uppercase letters (A–B) indicate significant differences between WS and RS starch under the same treatment. Treatment F(2,12) = 283.63, *p* < 0.001, η^2^p = 0.979, Starch F = 4.53, *p* = 0.055 ns, Interaction F = 5.49, *p* = 0.020.

**Table 7:** Docking score, energy profile, and binding modes of gluten subunits and starch fragments composite.

Samples (Best Binding Mode of Rigid-Body State)	Rigid-Body State		Emref State					
	High-Frequency Binding AAs of Best Mode [43] ^1^	Score ^2^	Score ^2^	bsa	desolv	elec	vdw	Total
Gliadin&Rice-amylopectin (1)	60–113	−12.1	−44.6	749.9	−3.8	−18.8	−37.1	−55.8
Gliadin&Rice-amylose (3)	61–101	−10.5	−42.5	510.0	−5.5	−17.4	−33.5	−50.9
Gliadin&Wheat-amylopectin (4)	40–59, 202–248	−7.1	−38.2	689.9	−4.4	−25.3	−28.8	−54.0
Gliadin&Wheat-amylose (1)	63–111	−12.0	−42.6	682.8	−6.3	−16.1	−33.1	−49.2
Glutenin&Rice-amylopectin (6)	111–120, 494–629	−13.8	−43.2	539.6	0.1	−35.8	−36.1	−71.8
Glutenin&Rice-amylose (4)	284–306, 323–368, 635–712, 826–848	−5.2	−47.5	657.0	−0.7	−45.1	−37.9	−82.9
Glutenin&Wheat-amylopectin (2)	110–276, 388–503, 753–848	−10.0	−45.2	616.4	−11.5	−36.5	−26.4	−62.9
Glutenin&Wheat-amylose (4)	284–301, 632–712, 826–848	−7.0	−35.2	304.4	−6.9	−17.0	−25.0	−41.9

Note: vdw (van der Waals) and elec (electrostatic) forces are favorable binding forces; desolv (desolvation) incurs energy consumption cost; bsa aids in characterizing the degree of hydrophobic burial; total represents the final overall binding energy, E_total_ = E_vdw(LJ)_ + E_elec(Coulomb)_ + E_desolv_ + E_BSA(penalty)_. ^1^ Ten thousand HADDOCK3 rigid-body docking poses were clustered by Ward’s linkage hierarchical agglomerative clustering and visualized via UMAP embedding (Python and R coding). Raw contact frequency (RawFreq) and energy-weighted frequency (EnergyWeightedFreq) at residue-level for each binding cluster were obtained from per-residue energy decomposition and compiled into a supplementary CSV dataset for identifying dominant protein–starch binding AAs and glucose. ^2^ This score represents the optimal binding conformation with the lowest energy at the corresponding stage.

## Data Availability

The original contributions presented in this study are included in the article/Supplementary Materials. Further inquiries can be directed to the corresponding authors.

## Supplementary Materials

The following supporting information can be downloaded at: https://www.mdpi.com/article/10.3390/foods15183243/s1.
