# Supplementary material for: Sequential Ultrasound-Assisted Enzymatic Hydrolysis and Microwave Treatment of Gluten–Starch Complexes: Functionality, Volatile Profiles, In Vitro Immunoreactivity and Interactions
Source: Foods. 2026 Sep 14;15(18):3243. doi: 10.3390/foods15183243 (PMC13605809; doi:10.3390/foods15183243)
Supplement: Supplementary file 1 [file foods-15-03243-s001.zip › Supplementary Materials.pdf]

Supplementary Information

Sequential Ultrasound-Assisted Enzymatic Hydrolysis and Microwave Treatment of Gluten–Starch Complexes: Functionality, Volatile Profiles, In Vitro Immunoreactivity and Interactions

Zhenlong Chen [1,†], Dong He [2,†], Xia Zhang [1], Lin Li [1,3], Bing Li [1*] and Xinhui Xing [4,5*]

[1] *School of Food Science and Engineering, South China University of Technology, Guangzhou 510640, China*

[2] *School of Chemical Engineering and Light Industry, Guangdong University of Technology, No. 100 Waihuan Xi Road, Panyu District, Guangzhou 510006, China*

[3] *School of Chemical Engineering and Energy Technology, Dongguan University of Technology, College Road 1, Dongguan 523808, China*

[4] *Institute of Biopharmaceutical and Health Engineering, Tsinghua Shenzhen International Graduate School, Shenzhen 518055, China*

[5] *Key Laboratory for Industrial Biocatalysis, Ministry of Education, Institute of Biochemical Engineering, Department of Chemical Engineering, Tsinghua University, Beijing 100084, China*

** Correspondence: bli@scut.edu.cn (B.L.); xhxing@mail.tsinghua.edu.cn (X.X.)† These authors contributed equally to this work.*

Supplementary Figure:

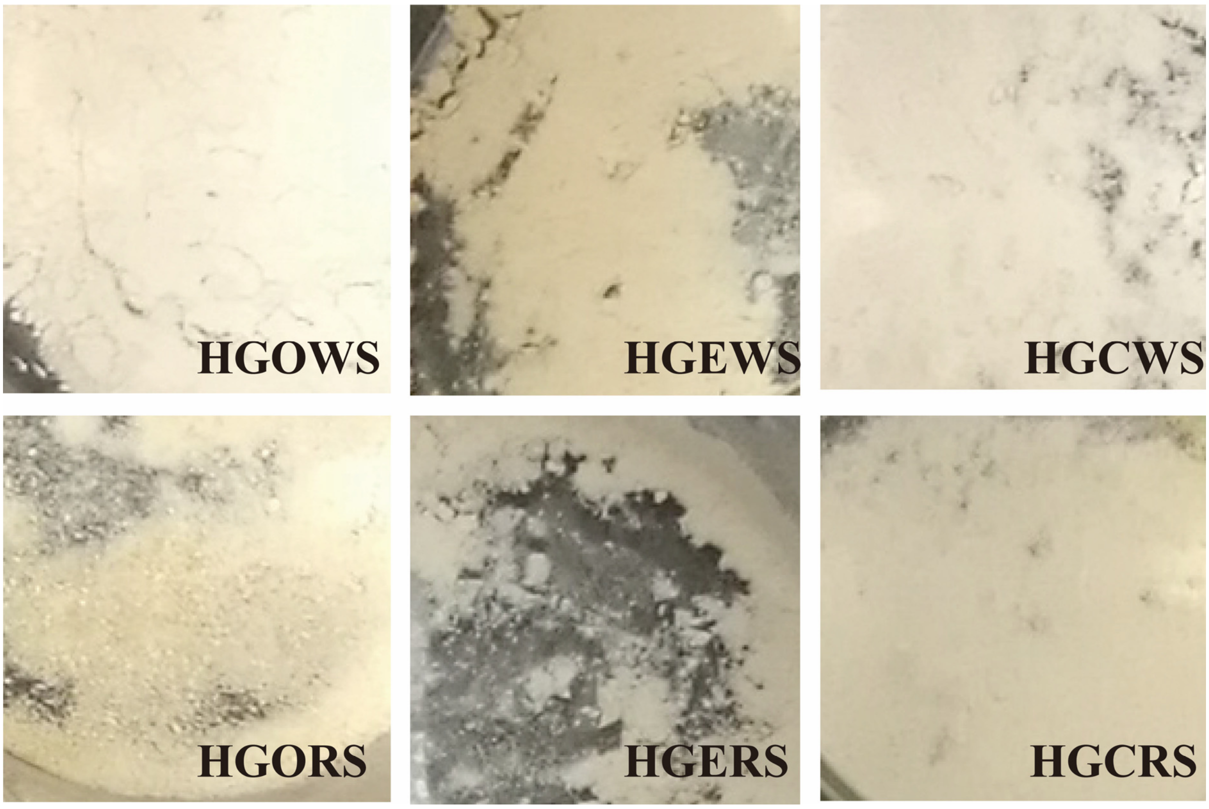

Figure. S1 The macroscopic solid powder of different MRCPs.

Supplementary Table:

Table S1 Tentative identification and odor descriptors of the ultra-fast GC-FID detected volatiles

Compound (CAS)	Compound descriptor
2-Acetyl-2-thiazoline (29926-41-8)	roasty, popcorn-like, baked meat-crust
Methional (3268-49-3)	boiled potato, brothy, malty, bread-crust
3-Methylbutanal (590-86-3)	malty, green, fruity, cocoa-like
2,3-Pentanedione (600-14-6)	buttery, creamy, sweet, dairy-like
Hexanal (66-25-1)	green, grassy, fatty, leafy
2-Methylthiophene (554-14-3)	sulfurous, onion-like, gassy
β-Ionone (14901-07-6)	woody, violet, floral, berry-like
Guaiacol (90-05-1)	smoky, phenolic, woody, toasty
Isoeugenol (97-54-1)	clove, spicy, warm, woody-floral
Methyl cinnamate (103-26-4)	sweet, balsamic, strawberry-fruity, cinnamon
Myristicin (607-91-0)	warm, spicy, balsamic, nutmeg-like
Methyl eugenol (93-15-2)	spicy, clove-like, warm-sweet, balsamic
Methanethiol (74-93-1)	sulfurous, cabbage, gassy; sweet-corn at low level
trans-4,5-Epoxy-(E)-2-decenal (188590-62-7)	metallic, blood-like, green, fatty-oxidized
2-Methyl-2-propanol (75-65-0)	camphoraceous, alcoholic, ethereal,

	solvent-like

Table S1 Tentative identification and odor descriptors of the ultra-fast GC-FID detected volatiles (continued)

Compound (CAS)	Compound descriptor
Skatole (83-34-1)	fecal/indole-like at high level; floral at high dilution
Bis(2-methyl-3-furanyl) disulfide (28588-75-2)	meaty, beefy, brothy, cooked-meat
Dimethyl trisulfide (3658-80-8)	sulfurous, alliaceous, cabbage, cooked-egg

Note: Compounds were tentatively identified by dual-column retention-index matching against the AroChemBase library (Alpha M.O.S.); only compounds with a library relevance score > 30 are listed. Odor descriptors were compiled from Fenaroli's Handbook of Flavor Ingredients [31] and The Good Scents Company information system (https://www.thegoodscentscompany.com/); descriptors of thermally generated character-impact volatiles were additionally interpreted following [32,33].

Table S2 Hydrogen bond network of molecular docking interaction between gluten protein subunits and starch fragments

Smaples	H-bond pairs of best model (A: Protein receptor residue, B: starch ligand residue)
Gliadin&Rice-amylose	GLN90(A)-GLC19(B), GLN90(A)-GLC21(B), GLN92(A)-GLC24(B), PRO93(A)-GLC24(B), ARG95(A)-GLC24(B)
Gliadin&Rice-amylopectin	LEU76(A)-GLC18(B), GLN79(A)-GLC19(B), GLN79(A)-GLC19(B), GLN77(A)-GLC20(B), GLN77(A)-GLC20(B), GLN79(A)-GLC20(B)
Gliadin& Wheat-amylopectin	GLN98(A)-GLC14(B), TYR100(A)-GLC14(B), GLN97(A)-GLC16(B), GLN97(A)-GLC16(B) GLN97(A)-GLC17(B)
Gliadin& Wheat-amylose	GLN65(A)-GLC11(B), ASN286(A)-GLC2(B), ASN286(A)-GLC3(B), ASN286(A)-GLC3(B), ASN286(A)-GLC3(B)
Glutenin&Rice-amylose	SER500(A)-GLC11(B), GLU27(A)-GLC17(B), GLU27(A)-GLC17(B), PRO119(A)-GLC18(B), THR499(A)-GLC21(B), THR499(A)-GLC21(B), TYR497(A)-GLC24(B)

Table S2 Hydrogen bond network of molecular docking interaction between gluten protein subunits and starch fragments (continued)

Smaples	H-bond pairs of best model (A: Protein receptor residue, B: starch ligand residue)
Glutenin&Rice-amylopectin	TYR703(A)-GLC11(B), GLY702(A)-GLC13(B), TYR703(A)-GLC13(B), TYR704(A)-GLC14(B), GLN358(A)-GLC17(B), GLY360(A)-GLC18(B), GLN358(A)-GLC20(B), PRO359(A)-GLC20(B), PRO359(A)-GLC20(B), GLN329(A)-GLC8(B)
Glutenin& Wheat-amylopectin	SER788(A)-GLC11(B), SER808(A)-GLC17(B), HIS810(A)-GLC17(B), TYR809(A)-GLC18(B), VAL811(A)-GLC19(B), VAL811(A)-GLC19(B), TYR784(A)-GLC23(B)
Glutenin& Wheat-amylose	TYR362(A)-GLC10(B), GLN358(A)-GLC11(B), GLN358(A)-GLC11(B), GLY360(A)-GLC11(B), GLY360(A)-GLC11(B), TYR362(A)-GLC7(B)
