# Supplementary material for: Sequential Ultrasound-Assisted Enzymatic Hydrolysis and Microwave Treatment of Gluten–Starch Complexes: Functionality, Volatile Profiles, In Vitro Immunoreactivity and Interactions
Source: Foods. 2026 Sep 14;15(18):3243. doi: 10.3390/foods15183243 (PMC13605809; doi:10.3390/foods15183243)
Supplement: Supplementary file 1 [file foods-15-03243-s001.zip › Supplementary_Note-Condition_screening_Methods_Response.pdf]

Supplementary Note

Sequential Ultrasound-Assisted Enzymatic Hydrolysis and Microwave Treatment of Gluten–Starch Complexes: Functionality, Volatile Profiles, In Vitro Immunoreactivity and Interactions

Zhenlong Chen [1,†], Dong He [2,†], Xia Zhang [1], Lin Li [1,3], Bing Li [1*] and Xinhui Xing [4,5*]

[1] *School of Food Science and Engineering, South China University of Technology, Guangzhou 510640, China*

[2] *School of Chemical Engineering and Light Industry, Guangdong University of Technology, No. 100 Waihuan Xi Road, Panyu District, Guangzhou 510006, China*

[3] *School of Chemical Engineering and Energy Technology, Dongguan University of Technology, College Road 1, Dongguan 523808, China*

[4] *Institute of Biopharmaceutical and Health Engineering, Tsinghua Shenzhen International Graduate School, Shenzhen 518055, China*

[5] *Key Laboratory for Industrial Biocatalysis, Ministry of Education, Institute of Biochemical Engineering, Department of Chemical Engineering, Tsinghua University, Beijing 100084, China*

** Correspondence: bli@scut.edu.cn (B.L.); xhxing@mail.tsinghua.edu.cn (X.X.)*

† These authors contributed equally to this work.

Preliminary Single-Factor (OFAT) Screening of the Microwave Maillard Conditions: Methods, Stepwise Logic, Final Settings and Reviewer Response (English, ready to paste)

A. Supplementary Methods

A1. Design rationale and coding

Before the formal experiment, a preliminary one-variable-at-a-time (OFAT) process screen was carried out to fix one mild, reproducible microwave window, so that the only deliberate factors in the formal study would be gluten treatment (GO, GE, GC) and starch type (wheat starch, WS; rice starch, RS). In this section and in Supplementary Figs. S1–S4, GO/GE/GC denote the untreated soluble gluten extract, the 6-h Protamex hydrolysate and the ultrasound-pretreated 6-h hydrolysate, respectively; WS and RS denote native wheat and rice starch. Microwave power, heating time, sample moisture and reaction mode, gluten:starch ratio and ambient relative humidity (RH) were varied in turn. Three reaction modes were distinguished throughout: (i) fully dry or moisture-conditioned powder heated by microwave (the route finally adopted); (ii) a dilute 5 mg/mL aqueous dispersion heated by microwave (microwave-wet mode); and (iii) an isothermal reference in which the 5 mg/mL dispersions were mixed 1:1 (v/v) and held in a 90 °C water bath for 2 h (water-bath wet mode). In every microwave run 2.0 g of blend was spread to the same thickness in an identical container; a 5-rpm rotating turntable and three independently prepared, randomly positioned replicates were used, and the endpoint surface temperature was checked (deviation < ±2 °C).

A2. Stepwise screen

The screen proceeded as a chain in which each run answered one open question and defined the variable tested next. Six screening runs across six rounds (Table S-N1). Each sub-run has its own macroscopic record; A294/A420 values that were assayed in the same measurement batch are presented together in the quantitative figures.

- Round 1 — Fully dry powder reaction speed at 600 W. A 1:1 gluten–wheat starch dry blend (60% RH) was heated at 600 W for 0, 5, 15, 25, 30 and 70 min. Colour barely changed up to 30 min and browning became visible only near 70 min. A dry system therefore reacted far too slowly, indicating that moisture was the limiting factor.
- Round 2 — Ratio and moisture level controls to avoid scorching. At 600 W for 10 min (85% RH), the ratios 1:1, 1:2 and 2:1 (gluten-wheat starch) and moisture levels of 10% and 50% were compared. The 1:1 blend with 0.5-part water and the 2:1:0 blend were already charred at 10 min, whereas 1:1 remained the most uniform. The ratio was fixed at 1:1 and only moderate moisture was retained.
- Round 3 — Possibility of lower power (360 W) workload for dry powder. A dense 2–75-min time scan of a 1:1 dry blend (73% RH) was run at 360 W (Supplementary Fig. S-N3, circular top views). Faint browning still required more than 60 min. Low-power heating of dry powder was judged impractical, directing the design back to 600 W with added moisture.

- Round 4 — Dry, conditioned or microwave-wet mode screening. At 360 W for 0–20 min (78% RH), fully dry powder (D), powder conditioned to 10% moisture and ground to confirm fully mixing in a mortar (M), and a 5 mg/mL microwave-heated aqueous dispersion (W) were compared. D stayed pale at 20 min, M began to yellow evenly at 15–20 min, and the microwave-wet dispersion remained almost colourless because dilution and evaporative heat loss limited the reaction. A moderately conditioned powder was therefore selected and the fully dry and dilute microwave-wet modes were set aside.
- Round 5 — Power confirmation and verification of Maillard chemistry (85% RH). A dry 1:1 blend was heated at 600 W for 0–32 min and a 5 mg/mL microwave-wet dispersion for 0–32 min, alongside a glycine–glucose positive Maillard control (all powders ground to confirm fully mixing after water addition). The 600-W conditioned/dry blend developed measurable browning earlier and more reproducibly than at 360 W, the microwave-wet dispersion stayed weak, and the positive control confirmed that the colour development was the Maillard reaction. Power was set to 600 W and moisture to the moderate 10–15% range, using the dry-conditioned powder route.
- Round 6 — Generalisability across treatments and starches. Conditioned (10% water, 79% RH, dry 1:1 blend) GO/GE/GC × WS/RS blends were preheated at 600 W for 5 min. And then a 5 min at 30 s intervals for 4 repeated run in total process has been identically conducted for all composites. All six combinations developed even, early-stage browning without scorching. A single long exposure was therefore replaced by short intermittent pulses to keep the reaction moderate and uniform. After detection of MRIPs and MREPs of 6 samples, the sample signal reached the edge of the UV detection linear range (A = 2). Therefore, one 5-min heating cycle was omitted (from 4 to 3) to avoid overheated or high values exceeding the reasonable linear detection range upon heating, which may be caused by the introduction of hydrolysis peptides fraction of peptide-enriched fractions~starch complexes from.

A3. Final microwave programme

GO/GE/GC were mixed with WS or RS at 1:1 (w/w). Distilled water was atomised onto the blend to ~15% moisture, verified with a halogen moisture analyser (HB43-S, Mettler Toledo, Switzerland), and the conditioned batter was equilibrated at 79% RH (Testo 608-H1, Testo, Germany). Heating used a calibrated multi-mode microwave reactor (MARS 6, CEM, USA): 600 W preheating for 5 min followed by three 5-min runs separated by 30-s intermissions. A 5-rpm turntable, 2.0-g loads spread to similar thickness and identical container, three randomly positioned replicates and temperature endpoint monitoring (deviation < ±2 °C) ensured uniform exposure. In the pilot A294/A420 data, 15% moisture with 30-s intermissions reached a measurable but still moderate reaction (10-times diluted samples), whereas 10% moisture with 30 s intermissions with 4 repeat 5 min heating run clearly over-reacted; this quantitative contrast, rather than sensory scoring, was the basis for the final setting. Microwave-treated composites or their water-soluble extracts were lyophilised.

A4. Readout and statistical scope

Absorbance at 294 nm (Maillard intermediate products, MRIPs) and 420 nm (late browning products, MREPs) was the quantitative readout; samples assayed in the same batch are plotted

together. Visual and olfactory inspection was used solely to discard charred, unevenly discoloured or off-odour samples; it was not a trained sensory assessment and entered no statistical analysis. This OFAT work was a process-setting pre-screen rather than a designed experiment, so no inferential statistics were applied to it. Formal two-way ANOVA (gluten treatment × starch type) was reserved for the six final composites.

B. Supplementary Note + stepwise logic table

Note. A preliminary OFAT screen was used to converge, run by run, on a single mild microwave window. Fully dry powder reacted too slowly at either 360 or 600 W (Rounds 1, 3); a high water fraction or a protein-rich ratio charred within 10 min (Round 2), whereas a dilute microwave-wet dispersion barely browned (Round 4). A 10%-conditioned powder was feasible at 600 W it developed an even, measurable Maillard reaction in a short time and a glycine–glucose positive control confirmed Maillard chemistry (Round 5). All six treatment × starch combinations browned evenly at 600 W without scorching (Round 6), so intermittent 600-W pulses were finally chosen to stay at the onset of browning and avoid hot spots. A294/A420 supplied the quantitative criterion; visual/olfactory inspection only excluded defective samples. The screened ranges, selected settings and rationale are summarised in Table S-N1, and the quantitative and visual records are given in Figs. S-N1–S-N10. No inferential statistics were applied to this pre-screen; the formal two-way ANOVA was used only for the six final composites.

Table S-N1. Run-by-run OFAT logic

Run	Main variable tested	Key observation	Decision for the next step
1	600 W, dry 1:1, 0–70 min	No colour to 30 min; browning only near 70 min	Dry powder too slow → add moisture
2	600 W, 10 min; ratio 1:1/1:2/2:1; 10%/50% water	1:1:0.5 and 2:1:0 charred; 1:1 most even	Fix 1:1; keep only moderate moisture
3	360 W, dry 1:1, 2–75 min	Faint browning only after >60 min	Abandon low-power dry heating
4	360 W, 0–20 min; dry / 10%-conditioned / microwave-wet	Dry stayed pale; microwave-wet nearly colourless; conditioned yellowed evenly at 15–20 min	Adopt moderately conditioned powder
5	600 W, dry & microwave-wet 0–32 min; Gly–Glc control (85% RH)	600 W conditioned blend browned earlier; wet stayed weak; control confirmed Maillard	Set 600 W and ~10–15% moisture
6	GO/GE/GC × WS/RS, 600 W, 5 min	All six blends browned evenly without scorching	Use short intermittent pulses; finalise protocol

Note: the question addressed, the observation made and it determined the next variable or the final setting.

C. Response to Reviewer Comment 18 (Lines 166–176)

Comment 18. The authors mention an "optimization of reaction conditions" but do not describe how this optimization was performed; the experimental design, variables evaluated and selection criteria should be included.

Response. We thank the reviewer for this request and agree that the screening procedure should be transparent. We no longer describe this stage as "optimization", which could imply a designed response-surface experiment; it was a preliminary one-variable-at-a-time (OFAT) process-setting screen, organised as six rounds (six screening runs in total). A complete subsection (Supplementary Methods A1–A4), a stepwise logic table (Table S-N1) and four supporting figures (Figs. S-N1~S-N10) have been added.

- Design. Microwave power (360 vs 600 W), heating time, sample moisture and reaction mode (dry, 10–15%-conditioned powder, microwave-heated 5 mg/mL dispersion, plus a 90 °C water-bath wet reference), gluten:starch ratio (2:1, 1:1, 1:2) and ambient RH (63–85%) were varied one at a time; each run fixed one factor and defined the next.
- Variables and selection criteria. A294 (MRIPs) and A420 (MREPs) were the quantitative readouts. A setting was retained only when it gave a measurable yet still moderate A294/A420 increase with an even appearance; formulations that charred, discoloured unevenly or developed off-odours were excluded by simple visual/olfactory inspection, which is explicitly stated not to be a sensory evaluation.
- Outcome. The screen converged on gluten:starch = 1:1, ~15% moisture, 79% RH equilibration and 600 W intermittent heating (5-min preheat plus three 5-min pulses with 30-s intermissions under turntable rotation and temperature control).

Statistical scope. Because this was a process-setting pre-screen, no inferential statistics were applied to it; the formal two-way ANOVA (gluten treatment × starch type) and all significance claims are restricted to the six final composites, as now stated in Methods 2.4 & 2.11 and also in Supplementary Methods A4.
